# *S*^3^Net: a Synthesis-Segmentation-Spiking Network for Alzheimer's disease detection and segmentation

**DOI:** 10.3389/frai.2026.1845114

**Published:** 2026-06-15

**Authors:** Gandham Dhanush Varmaa, Aravindkumar Sekar, Vemisetty Anshul, Ganugapenta V. Koushik Reddy, Praisy Evangelin A

**Affiliations:** School of Computer Science and Engineering, Vellore Institute of Technology, Chennai, India

**Keywords:** Alzheimer detection, Alzheimer segmentation, deep learning, generative adversarial network, spike neural network

## Abstract

Early and accurate detection of Alzheimer's disease (AD) from Magnetic Resonance Imaging (MRI) scans is crucial for clinical intervention. A novel *S*^3^Net, a Synthesis–Segmentation–Spiking Network, is proposed for this purpose. It integrates synthetic MRI generation, pathology-aware segmentation, and spike-based classification. The Synthesis Network uses a generative adversarial network framework. In this stage, original MRIs are fused with lesion-only patches from disease-relevant regions. This fusion helps preserve high-frequency pathological structures. The generator is trained with adversarial, L2, and Structural Similarity Index Measure (SSIM) losses. These losses ensure that the synthetic images remain realistic and structurally accurate. The Segmentation Network follows an encoder–bottleneck–decoder design with skip connections. It incorporates latent features from both the generator and the discriminator. A hybrid Dice–Binary Cross-Entropy loss is used to enable precise lesion delineation, even in sparsely annotated regions. For classification, a spiking network is employed. It takes fused segmentation and discriminator features and propagates them through Leaky Integrate-and-Fire neurons. This process captures temporal spike dynamics and supports low-power, event-driven computation. On the Open Access Series of Imaging Studies (OASIS) dataset, *S*^3^Net achieves an Accuracy of 95.1%, an F1-score of 93.0%, and an IoU of 82.6%. The proposed *S*^3^Net model outperforms other state-of-the-art methods, demonstrating its effectiveness and clinical viability for automated AD diagnosis.

## 1 Introduction

Alzheimer's disease (AD) is a progressive neurodegenerative disorder and also the most common form of dementia, characterized by progressive memory loss, cognitive decline, and behavioral impairment due to neuronal degeneration in the hippocampus and cortical regions in the brain (Zhou et al., 2025; Indhumathi and Palanivelan, 2025). More than 50 million individuals are affected by AD and this number will be tripled by the year 2050. This indicates the urgent need for early and accurate detection of disease (Turrisi et al., 2025). Structural Magnetic Resonance Imaging (sMRI) plays a vital role in AD research to visualize brain tissue atrophy and morphological alterations due to its non-invasive and superior spatial resolution; MRI helps to measure gray matter volume, hippocampal size, and cortical thickness, which are highly related biomarkers of cognitive decline (Zhou et al., 2025; Venkat et al., 2025). Magnetic Resonance Imaging (MRI)-based analysis supports identifying early neuroanatomical signatures before noticeable cognitive impairment and continues to be one of the widely used imaging biomarkers in AD disease (Kadri et al., 2025).

Recent progress in deep learning has led to a broad spectrum of MRI-based AD segmentation and classification methods. Many models focus on targeted anatomical structures; some examples are segmentation guided networks for the amygdala-hippocampi complex have shown improved discrimination between AD and control subjects by specifically isolating and analyzing these highly vulnerable regions in the medial temporal lobe regions (Zhou et al., 2025). Customised Convolution Neural Networks(CNN) architectures such as U-Nets and Alzh-Net-Style classifiers are used to analyze and extract hierarchical volumetric features from T1-weighted scans and achieve promising performance in AD diagnosis (Kadri et al., 2025; Venkat et al., 2025). Machine Learning pipelines that combine dimensionality reduction techniques with lightweight classifiers (like DRKPCA-ELM), also demonstrate competitive diagnostic accuracy, while significantly reducing the computational overhead (Haq et al., 2025). More complex designs are being developed that include attention mechanisms and transformer-based global context modeling, enabling the network to capture long-range dependencies that can effectively link changes across distant parts of the brain. It's essential for analyzing brain-wide atrophy patterns associated with a disease like Alzheimer's (Dakshinamoorthy et al., 2025; Islam et al., 2025). Beyond single modality MRI, multi-modal frameworks fuse structural MRI with Positron Emission Tomography (PET), demographic data, genetic risk scores, or plasma biomarkers using attention-based fusion, adaptive gating, or slice-level feature integration to get the complementary biological information (Huang et al., 2025; Turrisi et al., 2025). Generative Adversarial Network (GANs) and other related generators are used to augment the datasets, which are limited, and to translate between modalities to get better supervision and richer features, while these improve metrics and mitigate scarcity. Also, sometimes synthesized images can drift from ground truth if they are not constrained by structure-aware objectives.

Previous research studies show the rapid progress in deep learning based MRI analysis; however, several gaps still remain in Alzheimer's disease (AD) research. First, most segmentation models relies on Dice or cross—entropy losses without including structure-aware constraints such as pixel-wise (L2) with perceptual constraints (SSIM), which are helpful in preserving fine anatomical boundaries and inter-slice consistency, especially when the synthetic data are used essential for preserving anatomical boundaries and inter-slice consistency, particularly when segmentation is combined with generative processes (Zhou et al., 2025; Kadri et al., 2025; Indhumathi and Palanivelan, 2025). Secondly, multi-modal systems leverage attention and cross-scaling, but single modality MRI segmentation still lacks in fusing global embeddings with local details pathways via skip connections, limiting small-lesion/atrophy depiction (Kadri et al., 2025; Islam et al., 2025; Quek et al., 2023). Third, GAN-based synthesis improves the data diversity, but synthesis and segmentation are often treated independently; the absence of joint optimization with segmentation losses reduces the anatomical fidelity in the synthesized outputs (Huang et al., 2025; Venkat et al., 2025; Basanta-Torres et al., 2025; Hasan and Wagler, 2024). Fourth, Spiking Neural Network (SNN), which offers biologically plausible temporal encoding and energy-efficient learning, has seen limited application in MRI-based AD classification compared to established models like CNNs, Transformers, or Long Short-Term Memory Networks (LSTMs). Finally, many studies report only accuracy, F1-score, or Dice coefficient; it seldom report on statistical validation like Mathews Correlation Coefficient (MCC), SSIM, and *p*-value across multiple datasets to assert clinical-grade robustness and generalizability (Venkat et al., 2025; Sikka et al., 2025; Chen et al., 2025; Basanta-Torres et al., 2025; Zamani et al., 2022).

To overcome all these limitations, we propose a Synthesis-Segmentation-Spiking Network (*S*^3^Net) for MRI-based AD detection, which integrates generative reconstruction with structure-preserving segmentation and spiking neural classification into a unified framework. The proposed model combines pixel-level L2 loss and Structural Similarity (SSIM) loss in the segmentation path to enforce a structure-aware training, ensuring both intensity fidelity and perceptual consistency. This strategy explicitly preserves fine anatomical details that Dice-only segmentation models often miss (Zhou et al., 2025; Kadri et al., 2025; Indhumathi and Palanivelan, 2025). The segmentation module fuses the global embeddings with local spatial data via carefully designed skip connections to retain spatial details and enhance the detection of small lesions and subtle atrophy regions. Furthermore, end-to-end coupling of synthesis and segmentation stages guarantees that the generated or reconstructed MRI slices remain anatomically faithful to the segmentation target, addressing the “realism without correctness” problem observed in most of the conventional GAN-based augmentation (Venkat et al., 2025; Basanta-Torres et al., 2025). At the classification stage, Spiking Neural Networks (SNNs) use event-driven representations obtained from the segmentation module, offering strong classification with higher computational efficiency, an area that is unaddressed in prior MRI-based models. Finally, the proposed (*S*^3^-Net) model was rigorously evaluated using multiple MRI datasets, including OASIS, Alzheimer's cohorts, and general MRI datasets with F1, Dice, SSIM, and MCC, where the statistical significance (*p*-value) demonstrates consistently greater robustness, generalizability, and clinical reliability in line with recent systematic reviews and transfer-learning research.

The key contributions of this article are summarized as follows:

- A novel Synthesis-Segmentation-Spiking Network (*S*^3^Net) is proposed for MRI-based AD detection, segmentation, and classification.
- The network effectively reduces the losses to enhance pixel-wise reconstruction and structural consistency, improving segmentation quality and preserving fine anatomical details in MRI scans.
- Feature fusion of global and local embeddings (*f*_*g*_ + *f*_*d*_) along with skip connections in the segmentation module ensures retention of spatial information and boosts lesion detection performance across diverse MRI datasets.
- Extensive experimental evaluation shows that *S*^3^Net consistently outperforms existing state-of-the-art models in segmentation accuracy, anatomical detail preservation, and classification performance, demonstrating its suitability for clinical-grade AD detection

The organization of this article is as follows. Section 2 reviews the most relevant research works on AD diagnosis, including detection, segmentation, and classification. Section 3 describes the proposed synthesis–segmentation–spiking network (*S*^3^-Net) model in detail. Section 4 presents the experimental setup, including datasets, evaluation metrics, and implementation configuration. Section 5 provides the analysis and discussion of the experimental results. Finally, Section 6 concludes the study and highlights the key findings.

## 2 Related works

**Figure 1:**
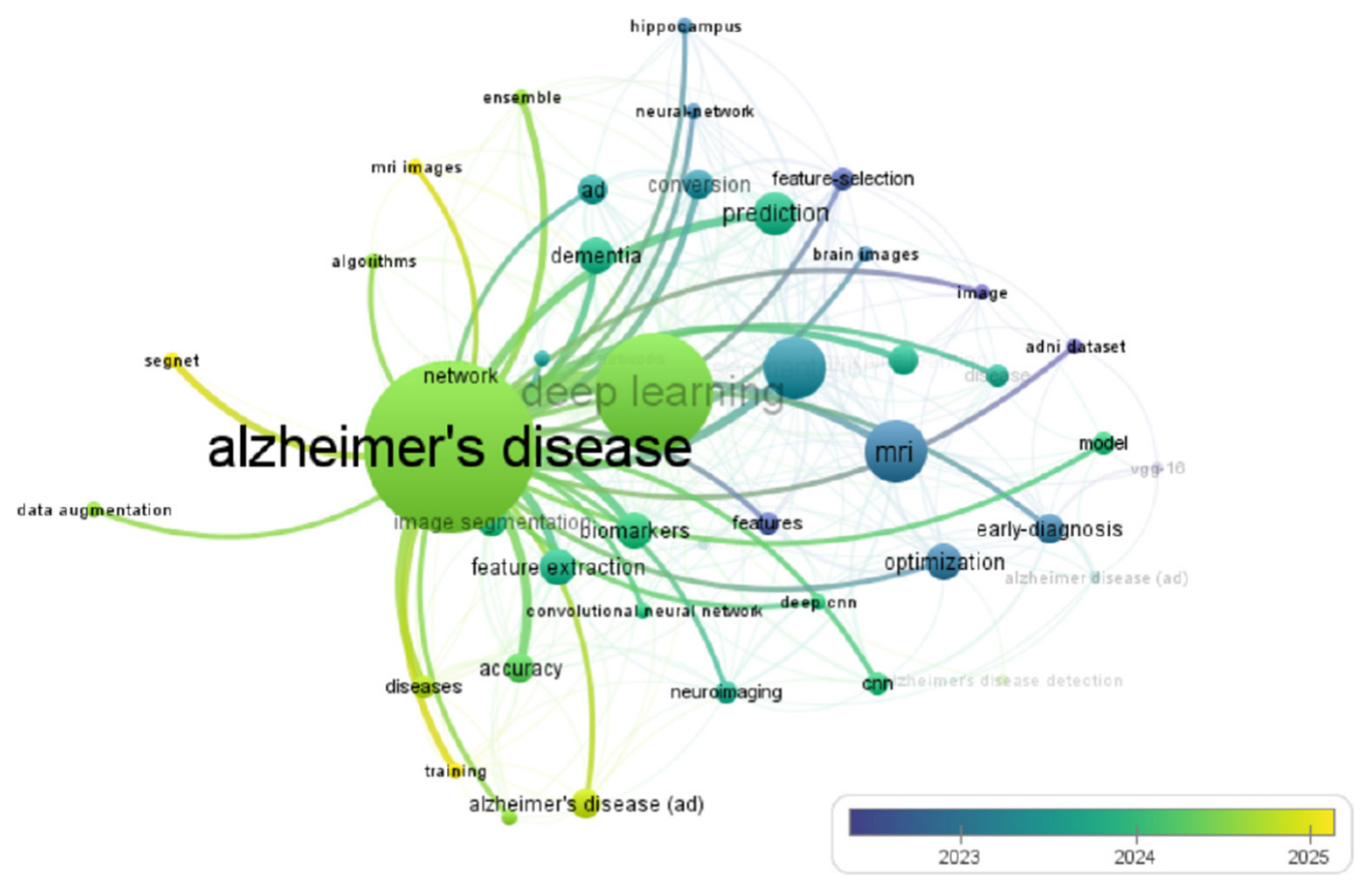
Keyword co-occurrence network of Alzheimer's disease research (202–2025), highlighting emerging trends in deep learning, MRI, and early diagnosis. Node size reflects keyword frequency; color shows topic evolution over time.

Alzheimer's disease (AD) detection has progressed from classic optimization-based segmentation toward advanced deep learning pipelines that analyze imaging data end-to-end. Early work combined bioinspired optimization methods with segmentation to better isolate brain regions such as gray and white matter, improving classification accuracy in later stages (Dakshinamoorthy et al., 2025). Hybrid loss–driven segmentation models further refined boundary detection, capturing subtle structural changes critical for early diagnosis (Indhumathi and Palanivelan, 2025). These outputs fed into deep architectures like multi-scale CNNs, which learned both fine- and coarse-grained features, boosting robustness against noise and class imbalance (Venkat et al., 2025). Lightweight multimodal CNN–LSTM frameworks soon emerged, reducing computational demands while maintaining high diagnostic accuracy, making real-time clinical use more practical (Haq et al., 2025). Systematic reviews have reinforced that pairing MRI-derived volumetric measures—especially in the medial temporal lobe—with neuropsychological assessments significantly improves early-stage detection over imaging alone (Bonarota et al., 2025). Figure 1 represents the network visualization of research trends related to AD using deep learning and MRI analysis (2023—2025). The size of each node represents the frequency of keyword occurrence, while the color gradient (blue to yellow) indicates the temporal evolution of topics from 2023 to 2025.

**Figure 2:**
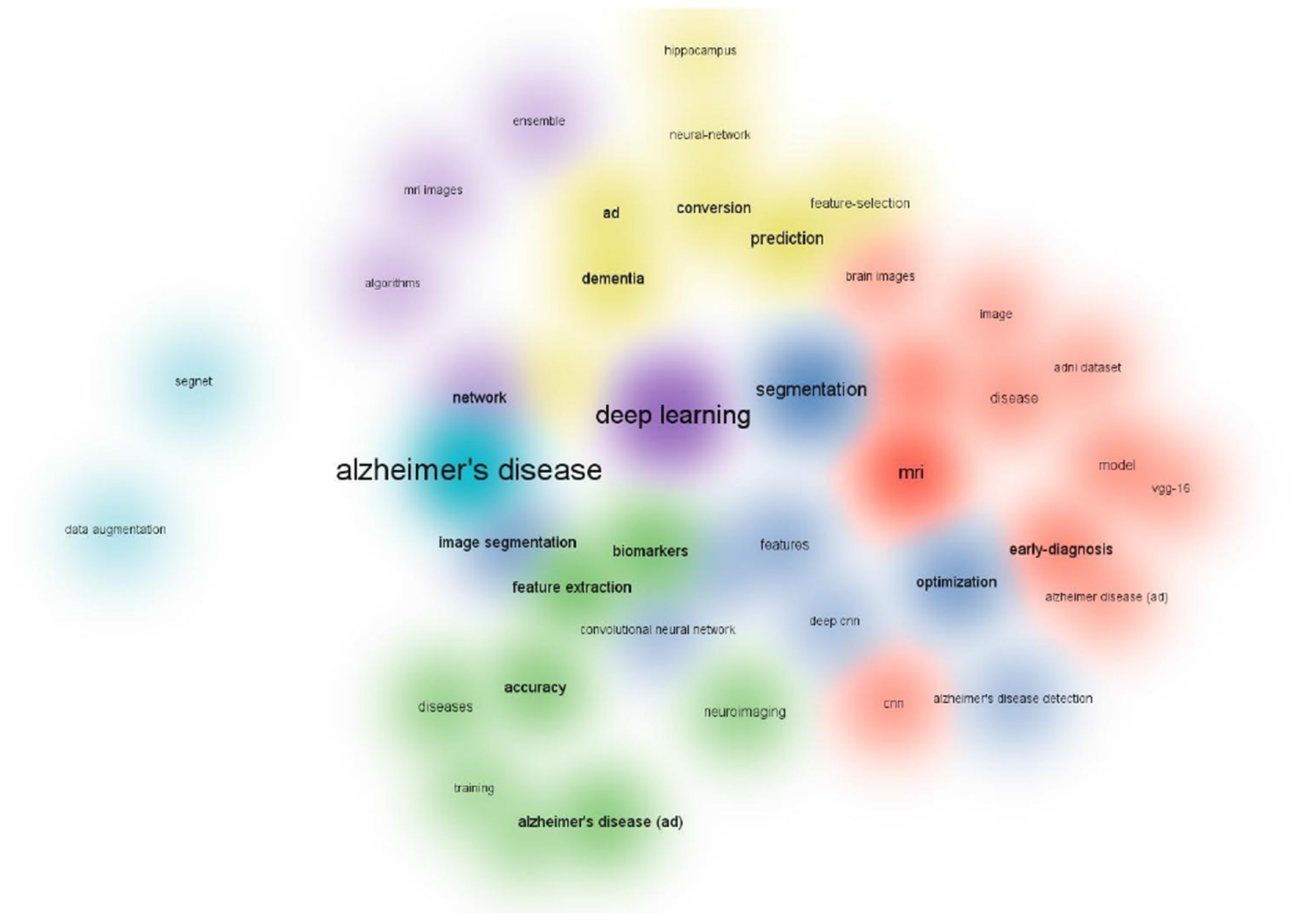
Cluster visualization of keywords related to Alzheimer's disease research using deep learning.

Advances in multimodal fusion brought structural and functional MRI together through spatio-temporal modeling and cross-modal attention, supported by missing-data recovery modules for improved robustness (Dakshinamoorthy et al., 2025). Biological validation came through transcriptomic studies revealing interneuron alterations linked to known AD biomarkers (Chen et al., 2025). Transfer learning and protocol-adaptive designs addressed variability across MRI scanners (Turrisi et al., 2025), while dual-decoder adversarial autoencoders paired with residual attention networks improved cross-protocol harmonization (Li et al., 2025). Multimodal feature fusion networks captured structural connectivity and temporal dynamics (Huang et al., 2025), enabling richer and more comprehensive brain representations that incorporate both micro- and macro-level patterns. Additionally, GAN-based pipelines began synthesizing PET from MRI or augmenting datasets to combat class imbalance, improving model generalization and sensitivity across different disease stages (Sikka et al., 2025). These GAN approaches not only reduce dependency on costly or less-available imaging modalities but also create diverse, high-quality synthetic samples, thereby enhancing the resilience, adaptability, and performance of deep learning systems in varied clinical and research environments. Figure 2 illustrates key research themes linking AD with deep learning, highlighting focus areas such as MRI-based diagnosis, segmentation, and disease progression prediction.

Recent developments have advanced these concepts further, incorporating richer clinical and biological information to enhance predictive accuracy. Biomarker-based frameworks have identified hippocampal volume and plasma p-tau as strong predictive markers for AD (Mohammed and Malhotra, 2025), while clinical research has established links between vascular health, glymphatic clearance, and AD risk (Wang et al., 2025). GAN-augmented training pipelines have addressed the limitations of small and imbalanced datasets by generating realistic synthetic samples for underrepresented AD progression stages, improving classification accuracy across multiple disease levels. In parallel, U-Net–based hippocampal localization models have demonstrated high precision in segmenting disease-relevant structures, enhancing both the segmentation stage and downstream classification performance (Pandey et al., 2025). Hybrid U-Net–GAN frameworks combine high-quality structural segmentation with targeted data augmentation, producing richer and more balanced training sets that improve resilience in deep learning models (Liu et al., 2025). The integration of these augmentation strategies has also enabled models to adapt more effectively to unseen patient data, narrowing the gap between experimental accuracy and deployment in hospital settings (Swarun Raj et al., 2026).

The most recent innovations leverage transformer–hybrid architectures (Kadri et al., 2025) for multimodal fusion and feature enhancement, and reinforcement learning–tuned CNNs optimized with adaptive strategies like CAdam (Chaudhari and Khot, 2025), addressing the core challenges of segmentation accuracy, modality integration, dataset harmonization, and robustness for real-world healthcare deployment. Together, these advances demonstrate a clear trajectory from segmentation-focused optimization toward highly integrated, augmentation-driven, and biologically validated deep learning systems (Yang et al., 2026). Such frameworks not only aim for high classification accuracy but also prioritize interpretability, enabling clinicians to trust model outputs. By incorporating domain-specific priors, adaptive learning strategies, and scalable cloud-based deployment pipelines, these systems hold the potential to deliver early, reliable, and clinically interpretable AD detection at a population scale, adaptable to both advanced research hospitals and resource-limited rural healthcare infrastructures (Zhao et al., 2026).

## 3 Proposed *S*^3^Net: Synthesis-Segmentation-Spiking Network model

**Figure 3:**
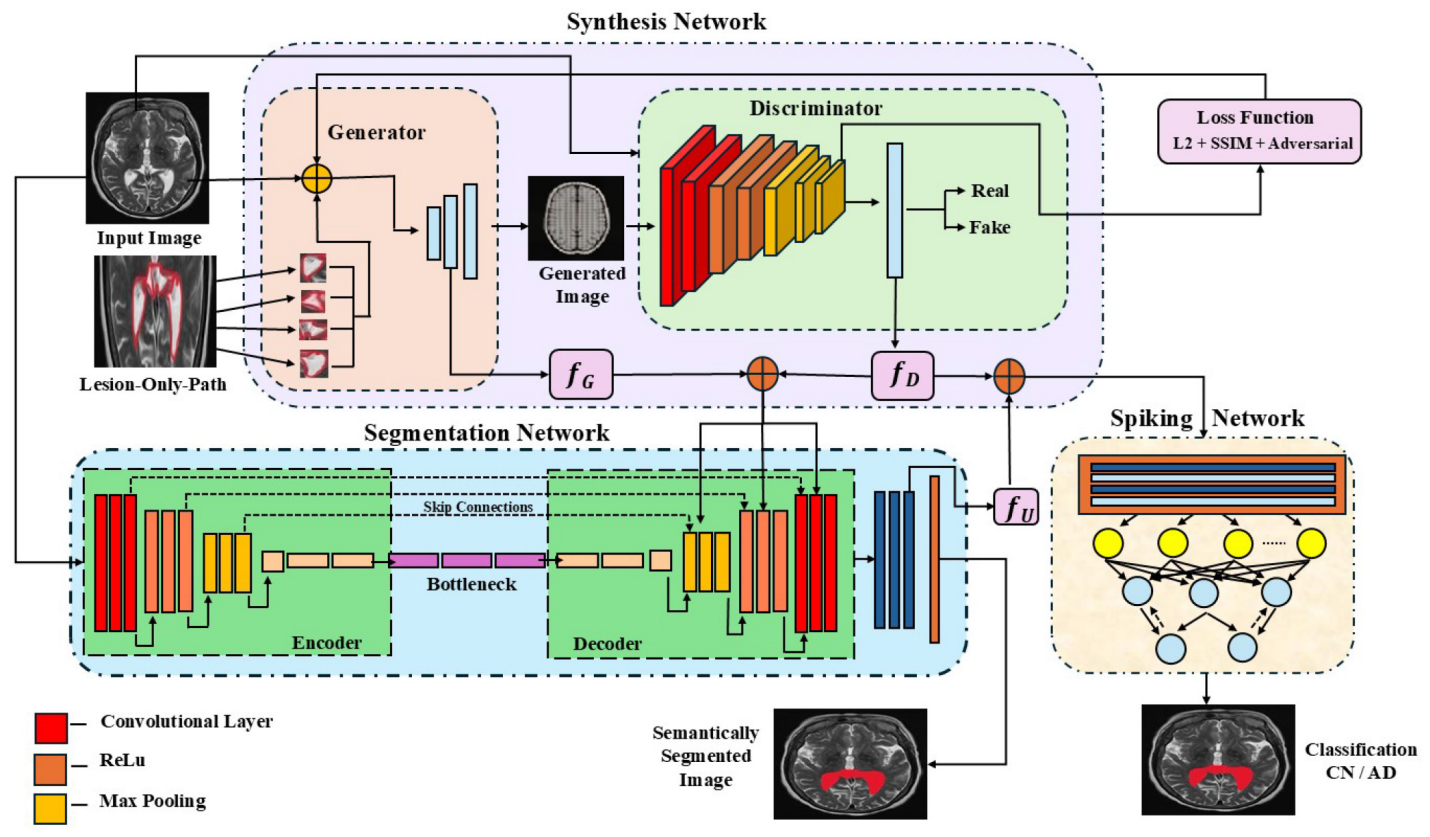
Illustrates the proposed *S*^3^Net: Synthesis-Segmentation-Spiking Network architecture.

Based on the above analysis, we propose a novel *S*^3^Net: Synthesis-Segmentation-Spiking Network for AD segmentation and classification. The proposed architecture integrates three sub-networks: a Synthesis Network, a Segmentation Network, and a Spiking Network. The Synthesis Network consists of a Generator and a Discriminator working together in an adversarial setup. The Generator creates synthetic AD images from MRI scans, while the Discriminator distinguishes real from generated images. The model is optimized with L2, SSIM, and Adversarial losses to enhance image quality and training performance. The Segmentation Network uses a contraction path to extract features, a Bottleneck layer to capture key information, and an expansion path to reconstruct the segmented image. It also incorporates skip connections to enhance feature flow and improve segmentation accuracy. Finally, the Spiking Network performs the classification of the segmented images into Cognitively Normal (CN) or AD categories. Figure 3 illustrates the overall architecture of the proposed *S*^3^Net: Synthesis-Segmentation-Spiking Network model. Table 1 presents the notation and its respective descriptions used.

**Table 1:** Notation and its description.

Notation	Description
*O*^*u*^, *O*^*v*^	Original MRI images used for generator (*u*) and discriminator (*v*) training batches.
$O_{\text{low}}^{\text{original}}$	Original MRI image dataset used for GAN training.
*LoP, LoP*_*i*_	Lesion-only patches extracted from MRI images; divided into four subregions (*i* = 1 to 4).
$G_{gen}$	Synthesized Alzheimer's MRI images generated by the GAN.
*f*_*g*_	Latent features extracted from the generator's penultimate layer.
*f*_*d*_	Latent features extracted from the discriminator's intermediate convolutional layer.
*I*_*orig*_	Original or synthesized MRI image used for segmentation.
*f*_*S*_	Segmentation feature extracted from the final fully connected layer of the segmentation network (input to SNN).
*I*_*i*_, *F*_*i*_	Intermediate feature maps from the *i*-th encoder layer.
*D*_*j*_, *U*_*j*_	Feature maps and upsampled activations in the *j*-th decoder layer.
*B*	Bottleneck feature representation in the encoder–decoder network.
*S*_*seg*_	Final MRI segmentation map output.
*S*_*n*_	Spiking neural network input derived from segmentation feature *f*_*S*_.
*f*_*D*_	Discriminator features used as input to the SNN.
*F*_*in*_	Integrated feature set combining *f*_*D*_ and *f*_*S*_ for SNN input.
*S*_*in*_(*t*)	Input spike trains encoded from *F*_*in*_ over time steps *t* = 1, …, *T*.
$V_{i}^{l}\left(t\right)$	Membrane potential of neuron *i* in layer *l* at time *t*.
$S_{i}^{l}\left(t\right)$	Spike output of neuron *i* in layer *l* at time *t*.
*V*_*th*_	Membrane firing threshold of spiking neurons.
λ	Leak constant controlling membrane decay rate.
*T*	Total simulation time (number of time steps).
*y*_*out*_	Predicted Alzheimer's class probabilities (AD / CN).
*y*_*true*_	Ground truth class label.
η	Learning rate for parameter updates.
*L*_*Gen*_, *L*_*D*_	Generator and discriminator loss functions in the GAN.
*L*_*adv*_	Adversarial loss for the generator.
*L*_*L*2_	Pixel-wise mean squared error loss.
*L*_*SSIM*_	Structural similarity loss for MRI quality preservation.
*L*_*seg*_	Combined segmentation loss using Dice and BCE terms.
*L*_*Dice*_	Dice loss component for overlap accuracy.
*L*_*BCE*_	Binary cross-entropy loss for segmentation.
*L*_*SpikeCE*_	Spike-based cross-entropy loss for SNN classification.
θ_*G*_, θ_*D*_, θ_*seg*_	Trainable parameters of the generator, discriminator, and segmentation network.
$W_{ij}^{l}$	Synaptic weight between neurons *j* and *i* in layer *l*.
σ(·)	Sigmoid activation function.
Softmax(·)	Softmax activation for output class probabilities.
*BN*(·)	Batch normalization operation.
*Conv*_3 × 3_, *ConvTrans*_3 × 3_	3 × 3 convolution and transposed convolution operations.
*MaxPool*(·)	Max pooling operation for downsampling.
*FC*_*final*_	Final fully connected layer for feature extraction in the segmentation network.

### 3.1 Pre-processing

**Figure 4:**
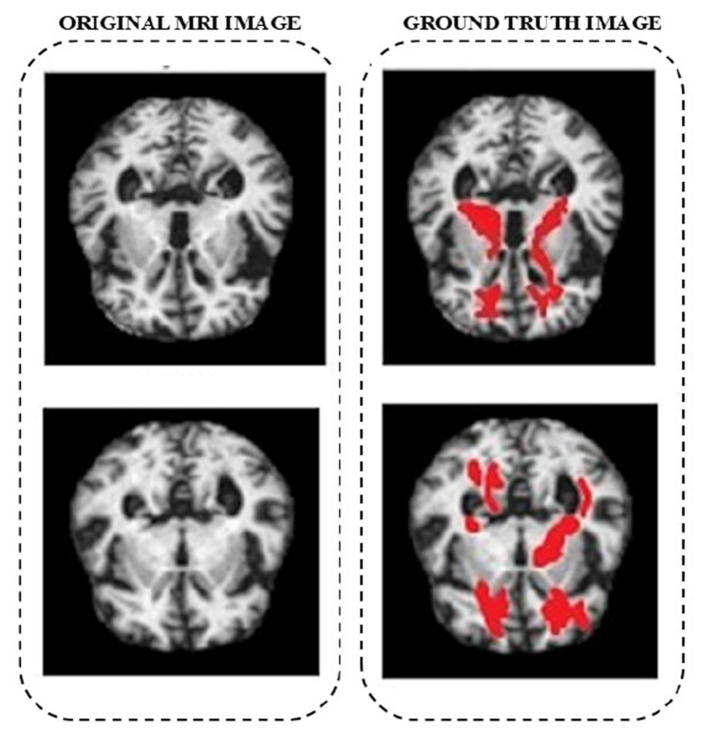
Illustrates original MRI images and corresponding ground truth annotations for Alzheimer's disease segmentation.

Prior to training, all MRI images are preprocessed to improve data quality, reduce noise, and ensure consistency across the datasets. Initially, the MRI images are resized, and intensity normalization is applied to standardize pixel distributions and minimize variations caused by different imaging conditions. To further enhance image quality, noise reduction techniques were incorporated to remove unwanted artifacts and improve structural clarity. In addition, image enhancement operations were performed to preserve important anatomical features relevant to AD analysis. The preprocessed MRI images were subsequently used as input to the proposed *S*^3^Net model for training. To avoid data leakage and ensure reliable experimental validation, subject-level splitting is adopted during dataset partitioning. The datasets were divided into 70% training data and 30% testing data for model training and evaluation. The ground truth masks used for segmentation represent anatomically relevant abnormal brain regions associated with AD progression. These regions were annotated using the corresponding lesion and pathological region labels available from the MRI datasets and preprocessing framework. During preprocessing, the annotated regions were spatially aligned with the MRI scans and converted into binary segmentation masks for supervised training. The segmentation network learns lesion-aware structural representations by comparing the predicted segmentation output with the corresponding ground truth masks. Figure 4 illustrates sample original MRI images and their corresponding ground truth annotations used for AD segmentation across different MRI samples.

### 3.2 Synthesized network

**Figure 5:**
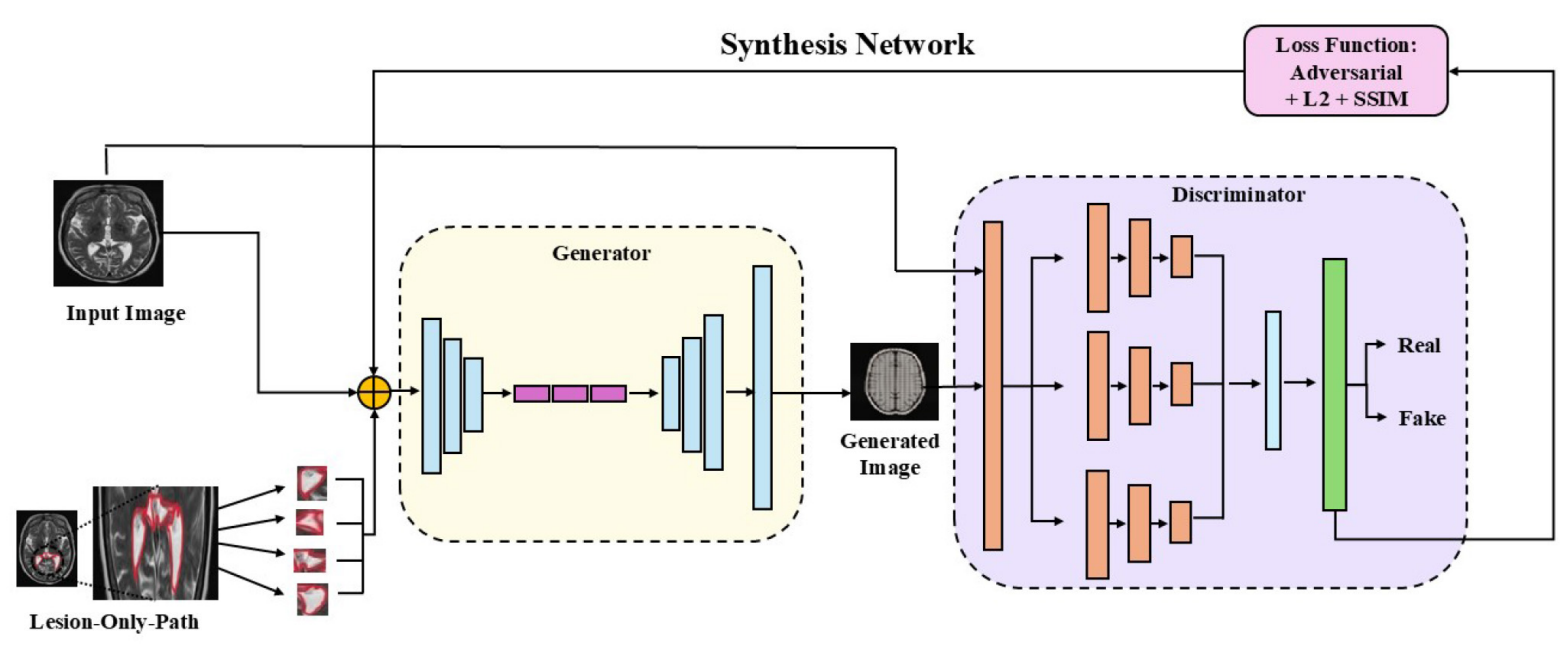
Illustrates the Synthesis architecture framework.

The proposed synthesis network generates Alzheimer's MRI images by combining a Generator *G* and a Discriminator *D* in a GAN framework. Algorithm 1 represents the stepwise procedure carried out for the synthesis network. The network receives two inputs: (i) the original MRI image and (ii) lesion-only patches (*LoP*) extracted from four disease-relevant brain regions. These lesion patches preserve high–frequency pathological structures and are fused with the input MRI image before synthesis. Figure 5 indicates the synthesized network architecture. Initially, the Synthesized Network utilizes the Equation 1.


$$\begin{array}{c} \underset{G}{\min}\underset{D}{\max}{𝔼}_{x\sim p_{\text{real}}}\left[\log D\left(x\right)\right]+{𝔼}_{z\sim p_{z}}\left[\log\left(1-D\left(G\left(z\right)\right)\right)\right] \end{array}$$ (1)

where *G* aims to synthesize realistic MRI images, and *D* aims to discriminate the real images from generated images. In our proposed approach, the Generator receives the fused input as highlighted in Equation 2.


$$\begin{array}{c} x^{u}=O^{u}+L^{i},\;L^{i}\in LoP \end{array}$$ (2)

where *O*^*u*^ represents the original MRI input and *L*^*i*^ denotes the lesion only patch from region *i*. Since the lesion-only patches (*LoP*) are of size 64 × 64 and the original MRI input *O*^*u*^ has dimensions 128 × 128, direct element wise addition is not performed initially. Each lesion-only patch is spatially aligned to its corresponding anatomical region within the original MRI image and then upsampled using interpolation to match the spatial resolution of 128 × 128. In addition, zero-padding can be applied to embed each lesion patch into its correct anatomical location, thereby generating a full-sized lesion representation. The four region-specific lesion patches are subsequently aggregated to form the final lesion map *L*^*i*^ with the same spatial dimensions as *O*^*u*^. This enables valid element-wise fusion in Equation 2.

Algorithm 1 Synthesis of Alzheimer's MRI images using GAN with generator, discriminator, and feature extraction for segmentation. 
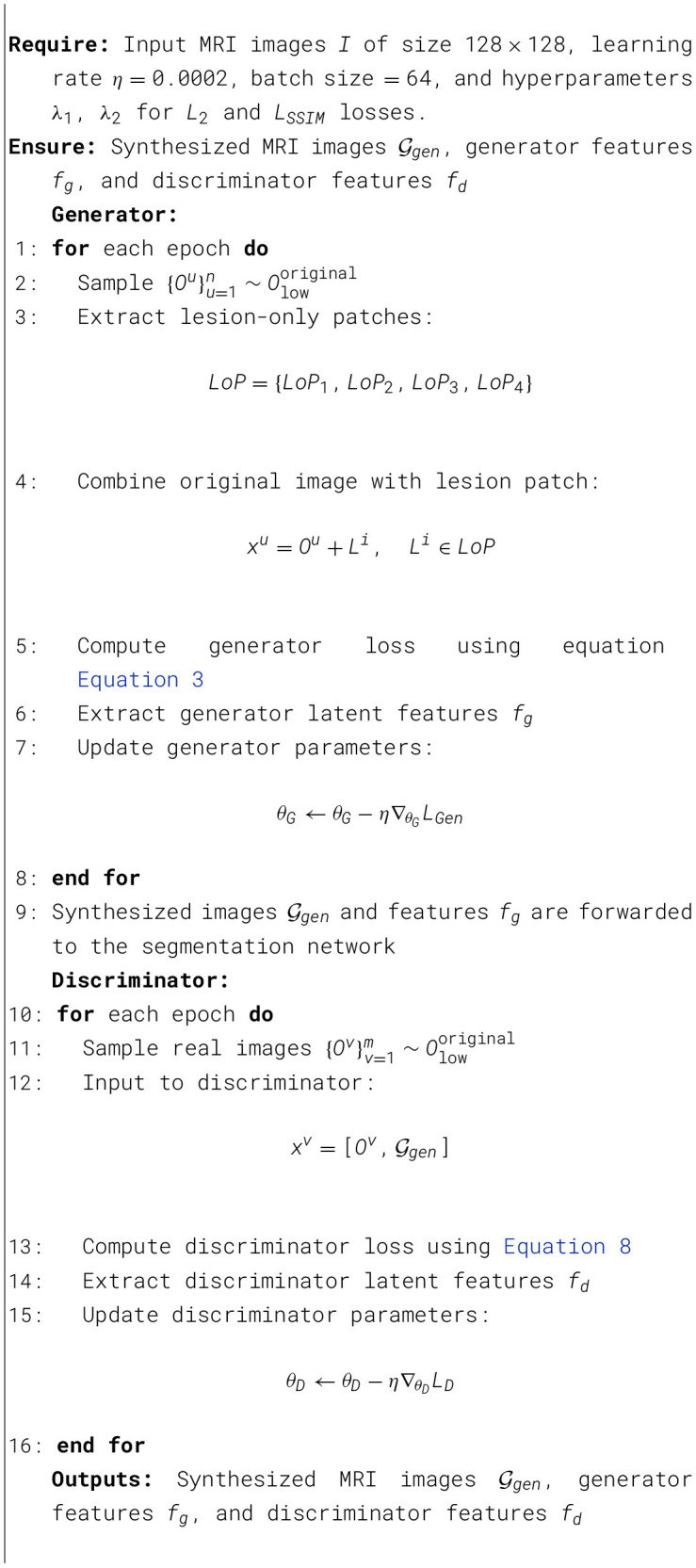

The Generator produces the synthesized Alzheimer MRI image *G*(*x*^*u*^), and the latent feature embedding *f*_*G*_ extracted from the penultimate fully connected layer is provided as the input to the segmentation network. The Generator is optimized by minimizing the following loss Equation 3.


$$\begin{array}{c} L_{\text{Gen}}=L_{\text{adv}}+\lambda_{1}L_{L2}+\lambda_{2}L_{SSIM} \end{array}$$ (3)

where *L*_adv_ represents the adversarial loss, *L*_*L*2_ denotes the pixel-wise reconstruction loss, and *L*_*SSIM*_ captures structural similarity between real and synthesized images. Their respective formulations are defined in Equations 4–6.


$$\begin{array}{c} L_{\text{adv}}=-𝔼\left[\log D\left(G\left(x^{u}\right)\right)\right] \end{array}$$ (4)

$$\begin{array}{c} L_{L2}=\frac{1}{N}\sum {\left(O-G\left(x^{u}\right)\right)}^{2} \end{array}$$ (5)

$$\begin{array}{c} L_{SSIM}=1-SSIM\left(O,G\left(x^{u}\right)\right) \end{array}$$ (6)

Thus, *L*_*L*2_ helps to improve the pixel-level accuracy between synthesized and original MRI images, while *L*_*SSIM*_ preserves the structural integrity of synthesized and original MRI images. The discriminator receives two inputs namely: (i) the original MRI image *O*^*p*^ sampled from the real data distribution, and (ii) the synthesized image *G*(*x*^*u*^), generated by the generator using the fused lesion region. These inputs are concatenated and fed into the Discriminator for feature extraction as formulated in Equation 7.


$$\begin{array}{c} x^{p}=O^{p},G\left(x^{u}\right) \end{array}$$ (7)

It extracts deep texture features of the lesion *f*_*D*_ and predicts probability scores for real and generated images. The discriminator loss is computed using Equation 8.


$$\begin{array}{c} L_{D}=-𝔼\left[\log D\left(O\right)\right]-𝔼\left[\log\left(1-D\left(G\left(x^{u}\right)\right)\right)\right] \end{array}$$ (8)

While training, *G* minimizes Equation 3 and *D* minimizes Equation 8. These loss functions guide the synthesis network to generate realistic MRI images with lesion details while preserving high structural similarity to the original images. Finally, the synthesized MRI *G*(*x*^*u*^) along with the feature embeddings *f*_*G*_ and *f*_*D*_ are passed to the segmentation network. This helps the network to segment the lesions more accurately by providing realistic and pathology-aware structural information.

### 3.3 Segmentation network

The synthesized MRI image *G*(*x*^*u*^), generated using the Synthesis Network and optimized using the generator loss in Equation 3, along with latent feature embeddings from the Generator (*f*_*G*_) and Discriminator (*f*_*D*_), is forwarded into the segmentation network. These embeddings retain pathology-aware structural cues learned during synthesis through adversarial loss (Equation 4), pixel reconstruction loss (Equation 5), and structural similarity loss (Equation 6). By incorporating them, the segmentation module receives similar structural and lesion-focused information, unlike conventional MRI-only segmentation approaches. The segmentation module follows an encoder–bottleneck–decoder architecture. In the encoder, convolution and max-pooling operations extract hierarchical texture features. Skip connections preserve spatial details by forwarding encoder feature maps *F*_*i*_ to the decoder. The bottleneck refines encoded feature representation using Equation 9.


$$\begin{array}{c} B={\text{LeakyReLU}}_{0.2}\left(BN\left(Conv_{3\times 3}\left(I_{enc}\right)\right)\right) \end{array}$$ (9)

During decoding, the up-sampled feature map is concatenated with the encoder output, the generator feature embedding, and the discriminator feature embedding using Equation 10.


$$\begin{array}{c} F_{dec}=\left[U_{j},F_{j},f_{G},f_{D}\right] \end{array}$$ (10)

**Figure 6:**
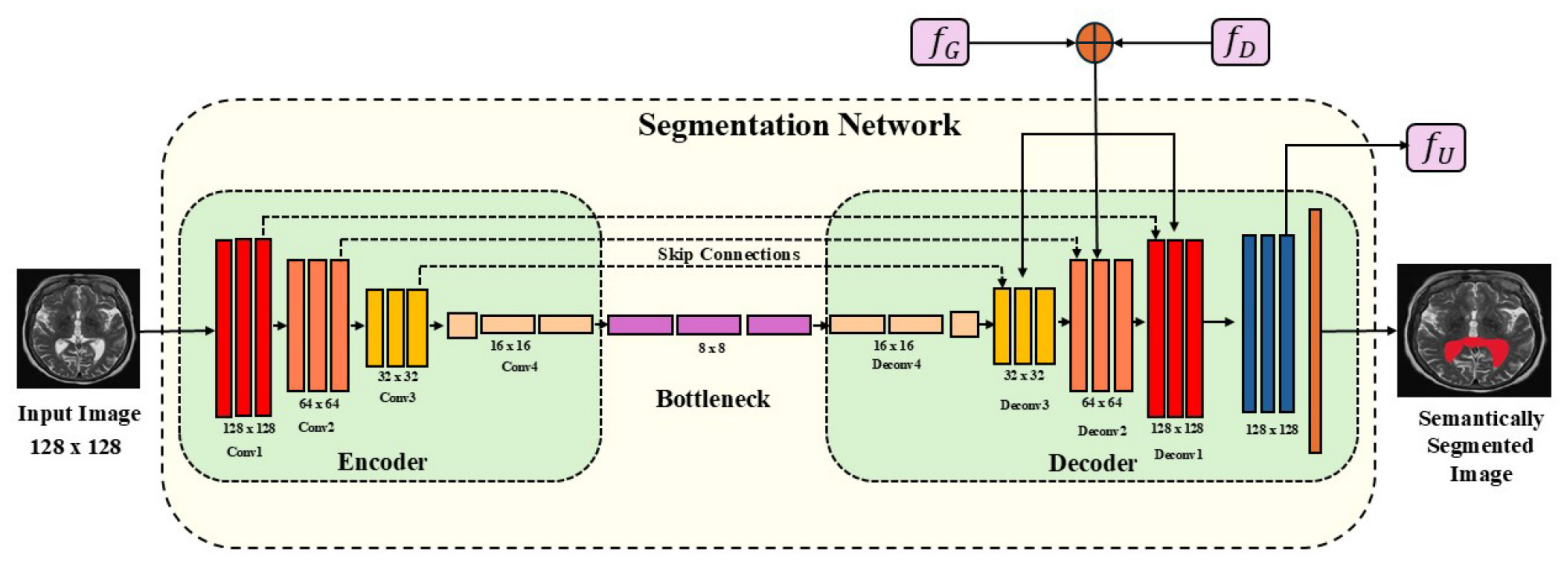
Illustrates the segmentation Network showing encoder-decoder architecture with skip connections and the integration of generator and discriminator features for enhanced MRI lesion segmentation.

The final segmentation mask is generated by applying a 1 × 1 convolution followed by a sigmoid function, as defined in Equation 11. This operation transforms the decoder output *D*_1_ into a probability map *S*_*seg*_. To achieve accurate lesion segmentation, the network is trained using a hybrid loss function shown in Equation 12. This hybrid formulation combines Dice loss and Binary Cross-Entropy (BCE). Dice loss evaluates the spatial overlap between the predicted mask (*P*_*m*_) and ground truth (*G*_*m*_), as expressed in Equation 13. This helps the network to better detect lesion boundaries, particularly in imbalanced datasets where lesion pixels are fewer than background pixels. BCE loss, defined in Equation 14, improves pixel-wise classification by penalizing incorrect predictions. The segmentation network parameters are optimized through gradient descent, as described in Equation 15. Once the segmentation output is generated, a high-level feature vector *F*_*s*_ is extracted from the final fully connected layer. This feature representation is then forwarded as input to the Spiking Neural Network for Alzheimer's classification, as shown in Equation 16. Algorithm 2 highlights the complete segmentation workflow. It highlights how the encoder–decoder architecture incorporates feature fusion from the Generator and Discriminator to improve lesion boundary detection and segmentation accuracy. Figure 6 represents the framework of the segmentation network.

Algorithm 2 Alzheimer's MRI segmentation using integrated generator (*f*_*g*_) and discriminator (*f*_*d*_) features within an encoder–decoder network. 
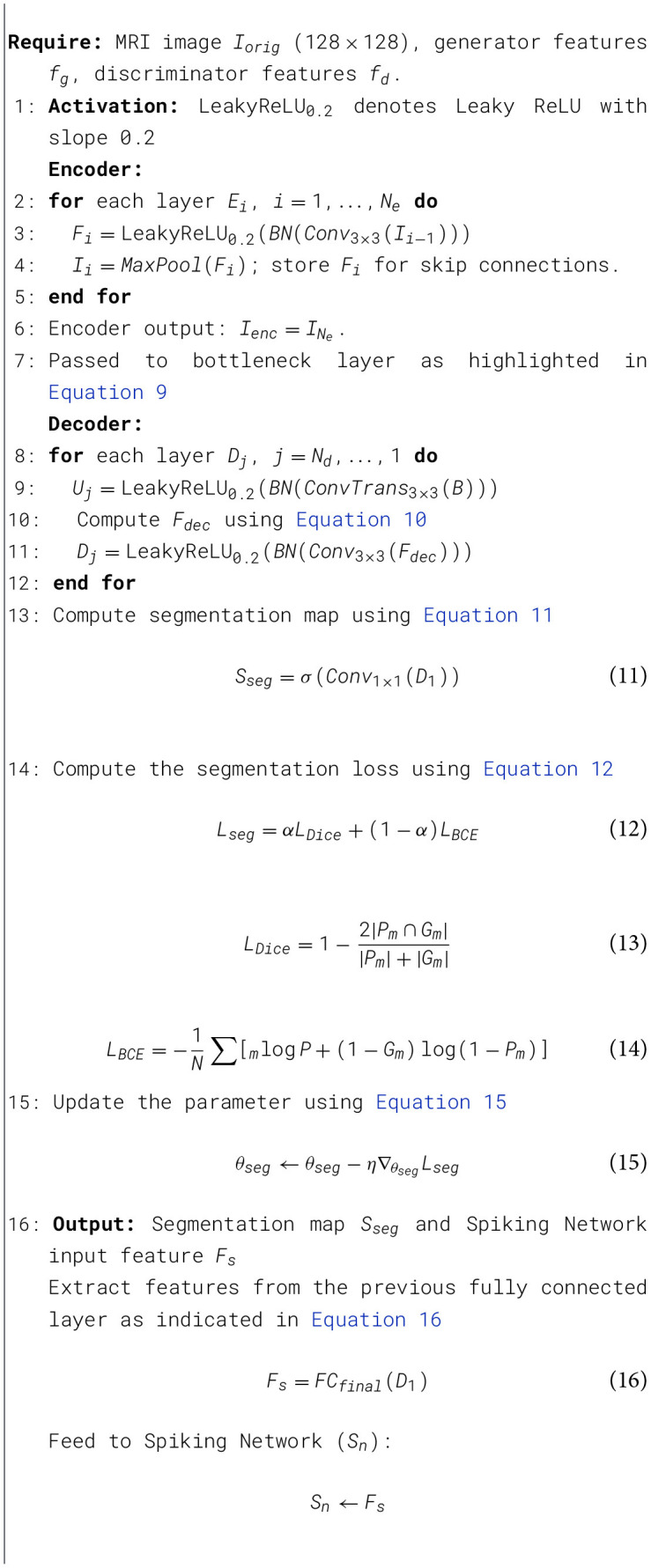

### 3.4 Spiking network

**Figure 7:**
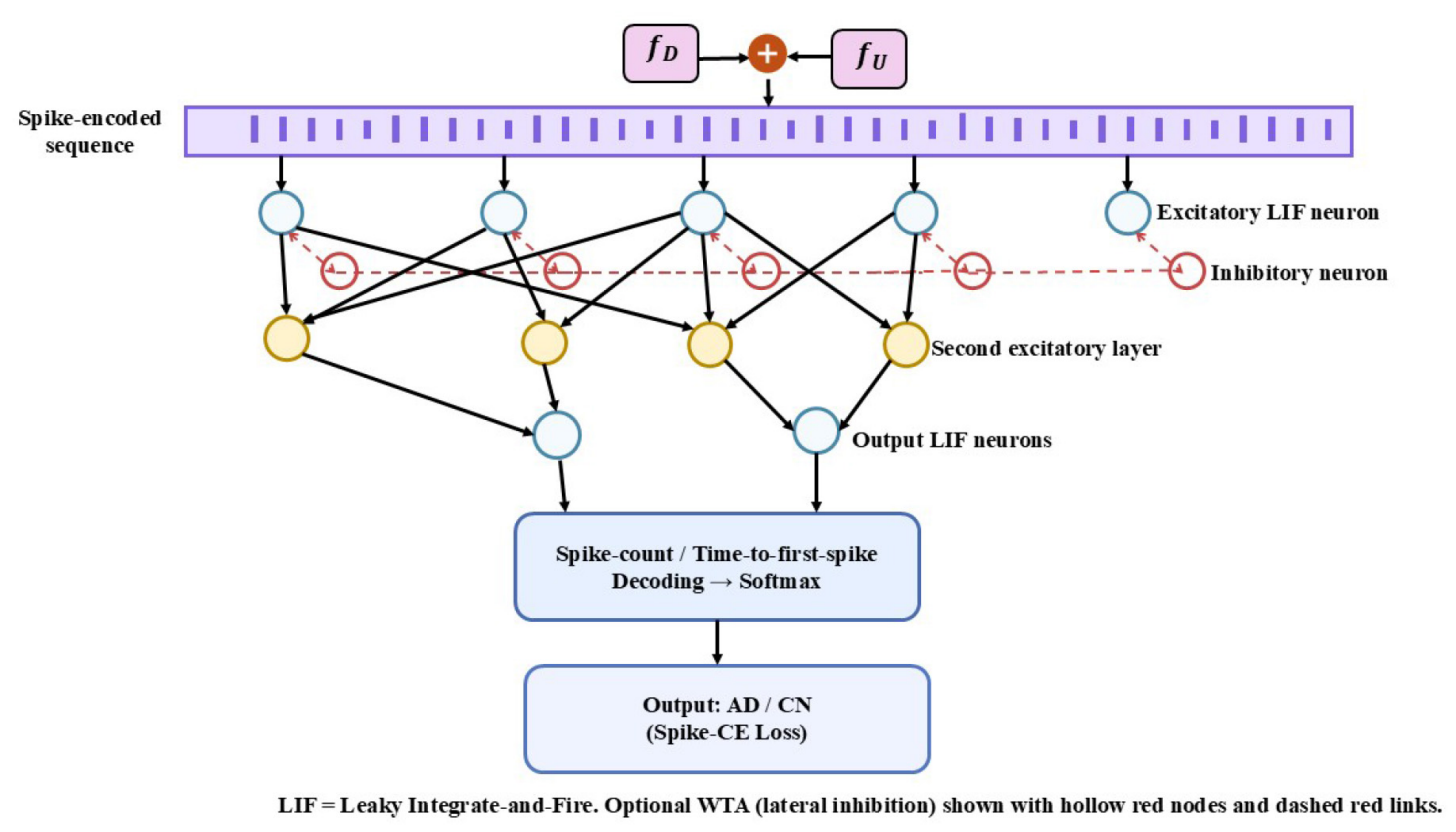
Spiking Network-based classifier using fused features (*f*_*D*_, *f*_*U*_), LIF neurons, and spike-based decoding for Alzheimer's disease or cognitively normal classification.

The final stage of the proposed *S*^3^Net model performs Alzheimer's classification using a Spiking Network (SN). Figure 7 indicates the spiking network framework. The fused feature vector *F*_*in*_ is obtained by combining the Discriminator feature embedding (*f*_*D*_) and the segmentation feature embedding (*f*_*S*_). This fusion operation is mathematically defined in Equation 26, ensuring that both structural information and lesion-specific patterns are retained. The fused vector is then converted into spike trains using temporal encoding, where the numerical value of each feature determines the spike firing rate, as formulated in Equation 27.

Once encoded, the spike sequence propagates through multiple layers of Leaky Integrate-and-Fire (LIF) neurons. At each simulation time step, the membrane potential of each neuron is updated using its previous potential and incoming weighted spikes, as governed by the LIF update rule in Equation 28. When the membrane potential exceeds a predefined firing threshold, the neuron emits a spike as described in Equation 29, after which the membrane potential is reset according to Equation 30. This allows the network to model biologically realistic temporal spike dynamics.

The spike activity of the output layer is aggregated over all time steps, and a Softmax activation is applied to produce final Alzheimer's classification probabilities (AD or CN), as expressed in Equation 31. During training, learning is performed using spike-based cross-entropy loss, shown in Equation 32, and the weights of the network are updated using gradient backpropagation through surrogate gradients (Equation 33). The SN captures temporal firing patterns instead of static activations, enabling low-power and event-driven computation suitable for neuromorphic and real-time medical diagnostic systems. Algorithm 3 illustrates the stepwise SNN-based Alzheimer's classification process. The features extracted from the discriminator and segmentation network are concatenated and encoded into spike trains. These spikes propagate through LIF neurons, and the accumulated spike activity is used to generate the final classification label more accurately. Table 2 presents the detailed architecture of the proposed *S*^3^Net framework for AD segmentation.

Algorithm 3 Spiking neural network for Alzheimer's classification. 
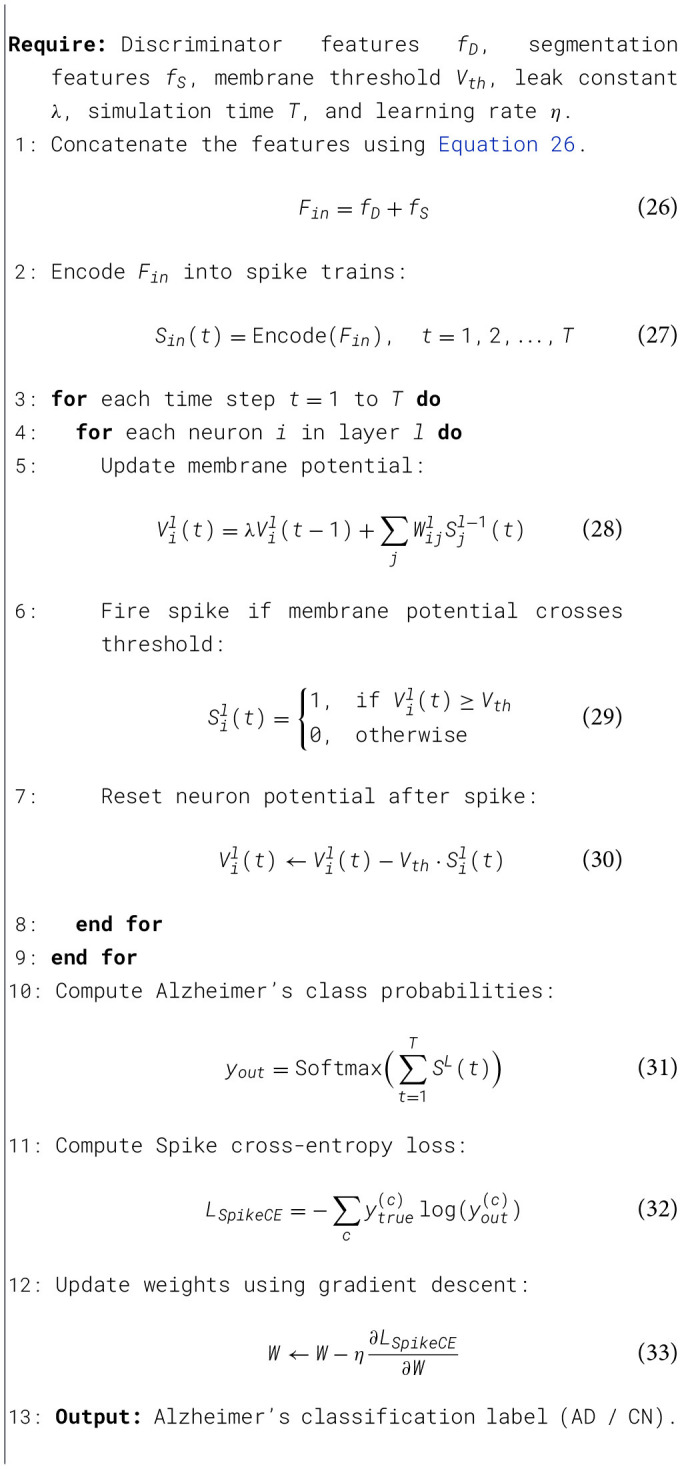

**Table 2:** Proposed *S*^3^Net network architecture.

Module	Operation	Input shape	Output shape
Input	MRI image	(128, 128, 1)	(128, 128, 1)
Synthesis input	Original MRI with lesion-only patch fusion	(128, 128, 1)	(128, 128, 1)
Generator	Conv2D-based synthesis network	(128, 128, 1)	Synthesized MRI
Generator feature	Latent feature extraction	Synthesized MRI	*f*_*g*_
Discriminator	Real/generated image discrimination	(128, 128, 1)	Real/Fake probability
Discriminator feature	Latent feature extraction	Intermediate discriminator layer	*f*_*d*_
Loss function	*L*_2_ + *L*_*SSIM*_ + *L*_*Adv*_ optimization	Generated MRI	Optimized synthesis output
Segmentation input	MRI image with *f*_*g*_ and *f*_*d*_ feature fusion	(128, 128, 1), *f*_*g*_, *f*_*d*_	Fused features
Encoder Block 1	Conv2D + ReLU + MaxPooling	(128, 128, 1)	(64, 64, 64)
Encoder Block 2	Conv2D + ReLU + MaxPooling	(64, 64, 64)	(32, 32, 128)
Encoder Block 3	Conv2D + ReLU + MaxPooling	(32, 32, 128)	(16, 16, 256)
Encoder Block 4	Conv2D + ReLU + MaxPooling	(16, 16, 256)	(8, 8, 512)
Bottleneck	Conv2D + ReLU feature refinement	(8, 8, 512)	(8, 8, 1, 024)
Decoder Block 1	UpSampling + Skip connection + *f*_*g*_/*f*_*d*_ fusion	(8, 8, 1, 024)	(16, 16, 512)
Decoder Block 2	UpSampling + Skip connection + *f*_*g*_/*f*_*d*_ fusion	(16, 16, 512)	(32, 32, 256)
Decoder Block 3	UpSampling + Skip connection + *f*_*g*_/*f*_*d*_ fusion	(32, 32, 256)	(64, 64, 128)
Decoder Block 4	UpSampling + Skip connection + *f*_*g*_/*f*_*d*_ fusion	(64, 64, 128)	(128, 128, 64)
Segmentation output	1 × 1 Conv2D + Sigmoid activation	(128, 128, 64)	*S*_*seg*_
Segmentation feature	Convolutional feature extraction	(128, 128, 64)	*f*_*U*_
SNN input	Feature fusion of *f*_*d*_ and *f*_*U*_	*f*_*d*_, *f*_*U*_	*F*_*in*_
Spiking network	Spike encoding + membrane update + Softmax	*F*_*in*_	AD/CN classification

## 4 Experiments

This section explains the experimental setup used to evaluate the proposed *S*^3^-Net model. First, we describe the benchmark MRI datasets used for training and validation. Next, we outline the evaluation metrics applied to measure both segmentation and classification performance. Finally, we present the implementation details, including the computational configuration and the overall training complexity.

### 4.1 Dataset

#### 4.1.1 OASIS MRI

The OASIS MRI dataset, sourced from the OASIS-1 collection for AD research, contains 80,000 MRI images with original slice dimensions of 496 × 248 pixels (Marcus et al., 2007). The dataset is categorized into four classes based on Alzheimer's severity: Non-Demented, Very Mild AD, Mild AD, and Moderate AD. Subject level splitting is adopted to avoid data leakage between training and testing samples. The dataset is divided into 70% training data and 30% testing data for experimental validation.

#### 4.1.2 Alzheimer's MRI dataset

The Alzheimer's MRI image has a pixel size of 128 × 128 (AbdelAziz et al., 2024). The dataset comprised both AD and Cognitively Normal.

#### 4.1.3 MRI dataset

It consists of 6,400 MRI images (Ullah et al., 2026). The dataset is divided into four classes: Non-Demented, Very Mild Demented, Mild Demented, and Demented. It includes both original scans and augmented images. The augmentation helps improve class balance and diversity -.

### 4.2 Evaluation metrics

We evaluated *S*^3^-Net using both segmentation and classification metrics. Segmentation performance was measured with Precision $\left(P_{S^{3}Net}\right)$, Recall $\left(R_{S^{3}Net}\right)$, F1-Score $\left(F1_{S^{3}Net}\right)$, Intersection over Union $\left(IoU_{S^{3}Net}\right)$, Dice $\left(Dice_{S^{3}Net}\right)$, Accuracy $\left(Acc_{S^{3}Net}\right)$, Matthews Correlation Coefficient $\left(MCC_{S^{3}Net}\right)$, and Structural Similarity Index $\left(SSIM_{S^{3}Net}\right)$. Each pixel was treated as a binary classification (lesion vs. non-lesion). Additionally, the quality of synthetic MRI images from the Synthesis Network was assessed using Fréchet Inception Distance (*FIDS*^3^*Net*) and Inception Score $\left(IS_{S^{3}Net}\right)$, where lower FID and higher IS indicate more realistic and diverse images. All metrics are computed using Equations 17–25.

- True Positive $\left(TP_{S_{Net}^{3}}\right)$ – Lesion pixels correctly predicted as lesion.
- True Negative $\left(TN_{S_{Net}^{3}}\right)$ – Non-lesion pixels correctly predicted as non-lesion.
- False Positive $\left(FP_{S_{Net}^{3}}\right)$ – Non-lesion pixels incorrectly predicted as lesion.
- False Negative $\left(FN_{S_{Net}^{3}}\right)$ – Lesion pixels incorrectly predicted as non-lesion.

- Precision $\left(P_{S_{Net}^{3}}\right)$
$$\begin{array}{c} P_{S_{Net}^{3}}=\frac{TP_{S_{Net}^{3}}}{TP_{S_{Net}^{3}}+FP_{S_{Net}^{3}}} \end{array}$$ (17)
- Recall $\left(R_{S_{Net}^{3}}\right)$
$$\begin{array}{c} R_{S_{Net}^{3}}=\frac{TP_{S_{Net}^{3}}}{TP_{S_{Net}^{3}}+FN_{S_{Net}^{3}}} \end{array}$$ (18)
- F1–Score $\left(F1_{S_{Net}^{3}}\right)$
$$\begin{array}{c} F1_{S_{Net}^{3}}=2\cdot \frac{P_{S_{Net}^{3}}\cdot R_{S_{Net}^{3}}}{P_{S_{Net}^{3}}+R_{S_{Net}^{3}}} \end{array}$$ (19)
- Intersection over Union $\left(IoU_{S_{Net}^{3}}\right)$
$$\begin{array}{c} IoU_{S_{Net}^{3}}=\frac{\left|P_{S_{Net}^{3}}\cap G_{S_{Net}^{3}}\right|}{\left|P_{S_{Net}^{3}}\cup G_{S_{Net}^{3}}\right|} \end{array}$$ (20)
- Dice $\left(Dice_{S_{Net}^{3}}\right)$
$$\begin{array}{c} Dice_{S_{Net}^{3}}=\frac{2TP_{S_{Net}^{3}}}{2TP_{S_{Net}^{3}}+FP_{S_{Net}^{3}}+FN_{S_{Net}^{3}}} \end{array}$$ (21)
- Accuracy $\left(Acc_{S_{Net}^{3}}\right)$
$$\begin{array}{c} Acc_{S_{Net}^{3}}=\frac{TP_{S_{Net}^{3}}+TN_{S_{Net}^{3}}}{TP_{S_{Net}^{3}}+TN_{S_{Net}^{3}}+FP_{S_{Net}^{3}}+FN_{S_{Net}^{3}}} \end{array}$$ (22)
- Matthews Correlation Coefficient $\left(MCC_{S_{Net}^{3}}\right)$
$$\begin{array}{r} MCC_{S_{Net}^{3}}=\frac{\left(TP_{S_{Net}^{3}}\cdot TN_{S_{Net}^{3}}\right)-\left(FP_{S_{Net}^{3}}\cdot FN_{S_{Net}^{3}}\right)}{\sqrt{\left(TP_{S_{Net}^{3}}+FP_{S_{Net}^{3}}\right)\left(TP_{S_{Net}^{3}}+FN_{S_{Net}^{3}}\right)}}\\ \cdot \frac{1}{\sqrt{\left(TN_{S_{Net}^{3}}+FP_{S_{Net}^{3}}\right)\left(TN_{S_{Net}^{3}}+FN_{S_{Net}^{3}}\right)}} \end{array}$$ (23)
- Fréchet Inception Distance (FID): The FID metric measures the similarity between the feature distributions of real and synthesized MRI images generated by the Synthesis Network in *S*^3^-Net, and it is computed using Equation 24.
$$\begin{array}{c} \text{FID}=\|\mu_{r}-\mu_{g}{\|}_{2}^{2}+\text{Tr}\left(\Sigma_{r}+\Sigma_{g}-2{\left(\Sigma_{r}\Sigma_{g}\right)}^{1/2}\right) \end{array}$$ (24)
  where μ_*r*_ and Σ_*r*_ are the mean and covariance of features extracted from real MRI images, and μ_*g*_ and Σ_*g*_ are the mean and covariance of features from synthesized images.
- Inception Score (IS): The Inception Score evaluates the diversity and quality of synthetic MRI images produced by the Synthesis Network, and it is computed in Equation 25.
$$\begin{array}{c} \text{IS}=\exp\left({𝔼}_{x\sim p_{g}}\left[D_{\text{KL}}\left(p\left(y|x\right)\|p\left(y\right)\right)\right]\right) \end{array}$$ (25)
  where *p*(*y*|*x*) is the conditional class distribution predicted by a pre-trained network for generated sample *x*, and *p*(*y*) = 𝔼_*x*_[*p*(*y*|*x*)] is the marginal distribution over all generated images.

### 4.3 Implementation details

The proposed *S*^3^-Net model is trained and evaluated on a high-performance workstation equipped with 2 × 24-core processors (48 cores total), 1 TB DDR5 system memory, and hybrid NVMe/SATA storage (1.9 TB NVMe for the operating system and 42 TB SATA for dataset storage). The system utilized 2 × NVIDIA H100 (80 GB) PCIe 5.0 GPUs, interconnected through NVIDIA NVLINK to enable high-bandwidth and parallel GPU execution. All experiments were conducted on Ubuntu 24.04 LTS using the PyTorch framework. The training configuration included input MRI images *I* of size 128 × 128, a learning rate η = 0.0002, a batch size of 64, and hyperparameters λ_1_ = 0.001 and λ_2_ = 0.01 for the *L*_2_ and *L*_*SSIM*_ loss components, respectively. The complete training process required 7 h and 52 min. During inference, the overall testing time across datasets is 41 s, where the model achieved an average segmentation time of 19.25 ms per image and a classification time of 1.4 ms per image. The computational complexity of the proposed *S*^3^-Net is highlighted in Table 3.

**Table 3:** Computational configuration used for training and evaluation of the proposed *S*^3^-Net.

Experimental configuration	Specification
CPU	2 × 24-core processors (Total: 48 cores)
Memory (system RAM)	1 TB DDR5
Storage	OS Drive: 1 × 1.9 TB NVMe PCIe SSD Data Storage: 3 × 14 TB SATA 6Gb/s (7.2K RPM)
GPU	2 × NVIDIA H100 Tensor Core GPUs, 80 GB HBM3 each (PCIe 5.0 × 16, passive cooling)
GPU interconnect	NVIDIA NVLINK Bridge
Operating system	Ubuntu 24.04 LTS
Deep learning framework	PyTorch (CUDA 12.2 + cuDNN optimized for H100)
Training time	7 h 52 min
Testing time	41 s
Average segmentation time per image	19.25 ms
SN classification time per image	1.4 ms

## 5 Results

This section presents the results of the proposed *S*^3^-Net model through both quantitative and qualitative evaluations. First, the segmentation performance is analyzed to determine the optimal configuration for accurate lesion region extraction. Next, the complete *S*^3^-Net framework integrating segmentation and classification is evaluated using multiple benchmark MRI datasets. Finally, the model is compared with recent state-of-the-art methods to demonstrate its effectiveness.

### 5.1 Comparative analysis of the proposed S^3^ Net on the OASIS MRI dataset

Table 4 presents the comparative analysis of the proposed *S*^3^ Net and its intermediate variants on the OASIS MRI dataset. The GAN (base model) achieves a precision of 0.724, a recall of 0.553, an F1-score of 0.627, an accuracy of 0.741, and MCC of 0.579. These values serve as the baseline for comparison. After incorporating the L2 loss, the GAN+L2 model improves to 0.781 in precision, 0.612 in recall, 0.686 in F1-score, and 0.792 in accuracy. This indicates better pixel-level reconstruction and more stable training. Adding the SSIM loss (GAN+SSIM) further enhances performance, achieving a precision of 0.816, recall of 0.678, F1-score of 0.741, and accuracy of 0.832. The improvement shows that SSIM helps preserve structural details in the MRI images.

**Table 4:** Comparative analysis of results obtained for OASIS MRI dataset.

Model	Precision	Recall	F1-score	Accuracy	IoU	Dice	SSIM	MCC
GAN (base model)	0.724	0.553	0.627	0.741	0.482	0.650	0.602	0.579
GAN+L2	0.781	0.612	0.686	0.792	0.546	0.706	0.654	0.632
GAN+SSIM	0.816	0.678	0.741	0.832	0.594	0.745	0.689	0.675
Base Model+L2+SSIM	0.854	0.713	0.777	0.861	0.635	0.777	0.719	0.731
*f*_*g*_ +*f*_*d*_ + without skip connections	0.872	0.782	0.825	0.883	0.672	0.804	0.752	0.775
*f*_*g*_ +*f*_*d*_ + with skip connections	0.915	0.884	0.899	0.923	0.739	0.850	0.809	0.841
*f*_*g*_ +*f*_*d*_ + with skip connections + MLP	0.928	0.892	0.910	0.931	0.756	0.867	0.821	0.854
Proposed *S*^3^ Net	0.942	0.918	0.930	0.951	0.826	0.905	0.879	0.893

**Figure 8:**
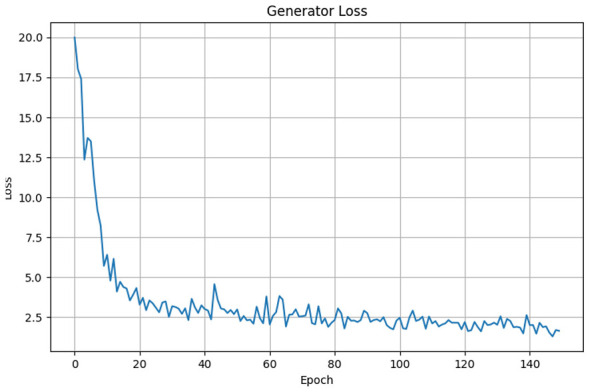
Illustrates the generator loss vs. epochs.

**Figure 9:**
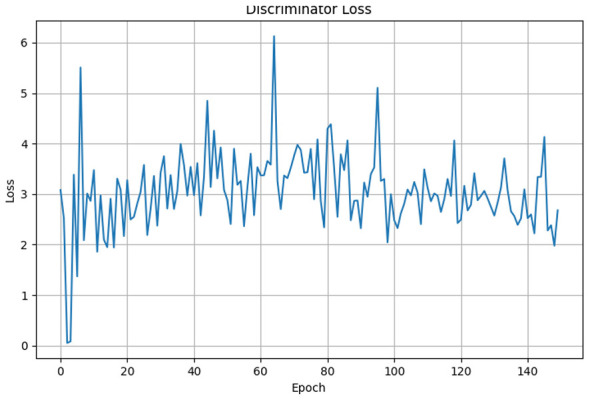
Illustrates the discriminator loss vs. epochs.

Further integrating both L2 and SSIM losses in the Base Model+L2+SSIM yields a precision of 0.854, a recall of 0.713, F1-score of 0.777, and an accuracy of 0.861. This demonstrates the complementary effect of pixel-wise and perceptual losses, improving segmentation quality. Introducing feature fusion with *f*_*g*_ + *f*_*d*_ and the U-Net architecture, without skip connections, improves performance. It achieves a precision of 0.872, a recall of 0.782, an F1-score of 0.825, and an accuracy of 0.883. Incorporating skip connections improves results further. The *f*_*g*_ + *f*_*d*_ + U-Net with skip connections records a precision of 0.915, a recall of 0.884, F1-score of 0.899, and an accuracy of 0.923. This confirms that skip connections help preserve spatial information during decoding. Figures 8, 9 show that the generator loss decreases steadily, while the discriminator loss fluctuates over 150 epochs, indicating stable convergence and adversarial balance during GAN training.

**Figure 10:**
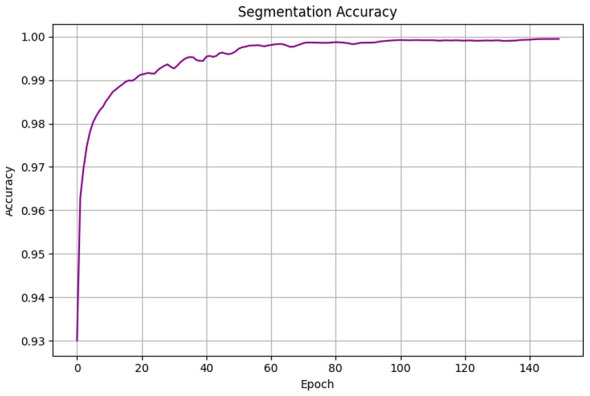
Illustrates the accuracy of the segment network.

**Figure 11:**
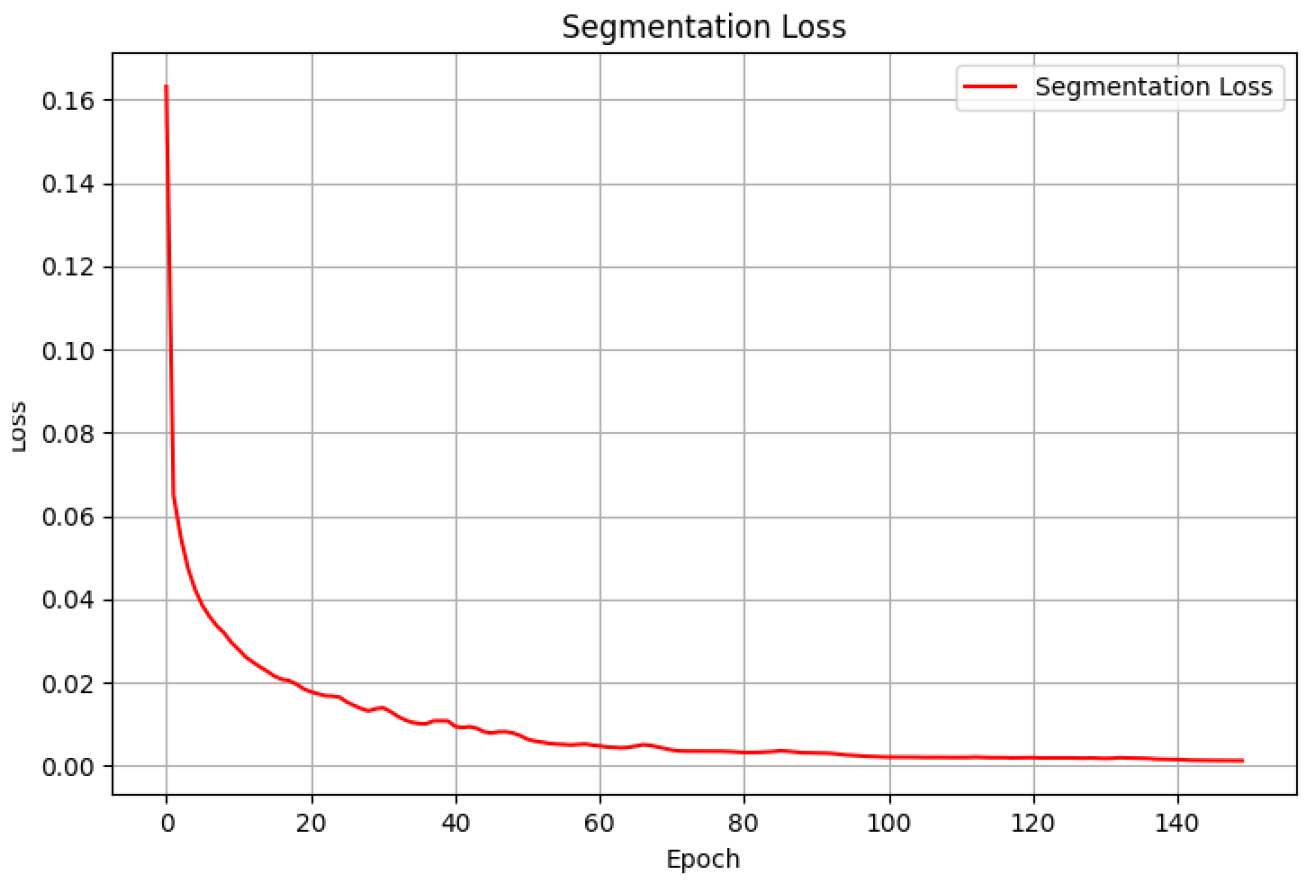
Illustrates the loss of the segment network.

**Figure 12:**
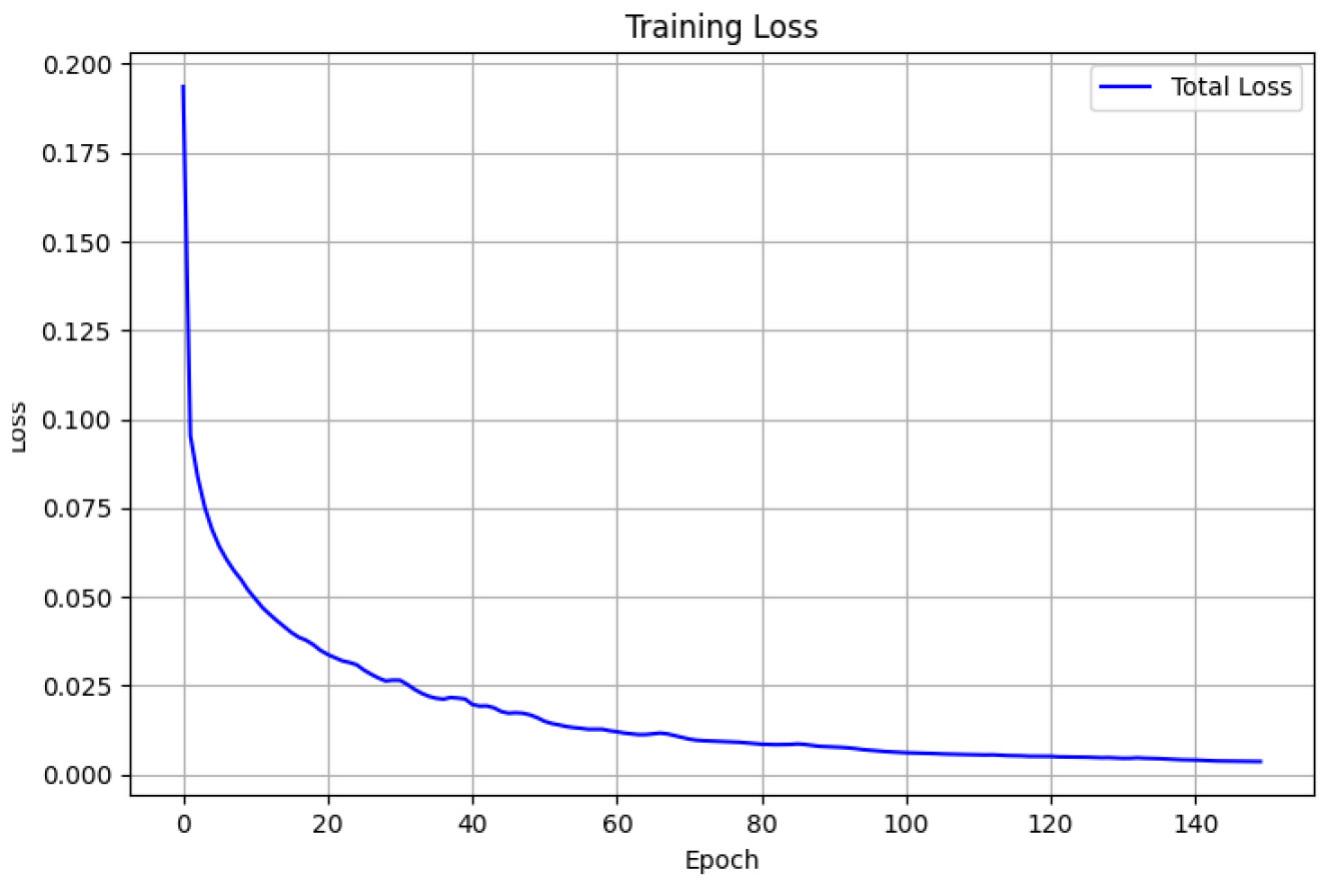
Illustrates the loss of the spiking network.

The proposed *S*^3^Net achieves the best results among all models. It attains a precision of 0.942, recall of 0.918, F1-score of 0.930, and accuracy of 0.951. Compared to the base GAN, it improves F1-score by 30.0%, accuracy by 21.0%, and MCC by 31.7%. The Dice coefficient (0.905) and SSIM (0.879) confirm its strong ability to preserve anatomical details and structural consistency. Replacing the Spiking Neural Network with an MLP classifier in the *f*_*g*_ + *f*_*d*_ + U-Net with skip connections framework achieves a precision of 0.928, recall of 0.892, F1-score of 0.910, accuracy of 0.931, and MCC of 0.854, demonstrating that the proposed Spiking Network further improves discriminative learning and classification performance over conventional MLP-based classification. Figures 10, 11 depict the segmentation network's accuracy and loss during training. Figure 12 shows the spiking network's loss.

We also evaluate the quality of synthetic MRI images from the Synthesis Network using Fréchet Inception Distance (FID) and Inception Score (IS) (Equations 24, 25). The *S*^3^Net achieves an FID of 18.6 ± 0.3 and an IS of 4.05 ± 0.08. These results indicate high fidelity and diversity of the synthesized images. Lower FID and higher IS indicate synthesized MRIs that are realistic and diverse. This contributes to the robustness and accuracy of both segmentation and classification tasks.

### 5.2 Analysis of results obtained for Alzheimer's MRI Dataset

Table 5 highlights the performance of the proposed *S*^3^ Net and its variants on the Alzheimer's MRI dataset. The GAN base model shows limited segmentation capability. It achieves an F1-score of 0.603 and accuracy of 0.715. These results reflect weak feature representation and unstable training. Introducing the L2 loss improves pixel-wise reconstruction. The GAN+L2 variant reaches an F1-score of 0.666 and accuracy of 0.774. This indicates more stable convergence and enhanced image recovery. Adding SSIM loss enhances structural preservation. The GAN+SSIM model attains an F1-score of 0.716 and accuracy of 0.809. The Base Model with both L2 and SSIM losses further improves performance. It achieves an F1-score of 0.760 and accuracy of 0.844. This demonstrates the complementary effect of combining pixel fidelity and structural consistency losses. Incorporating feature fusion through *f*_*g*_ + *f*_*d*_ + U-Net without skip connections leads to better feature representation. This variant achieves an F1-score of 0.801 and an accuracy of 0.861. The results suggest that effective integration of global and local features enhances segmentation accuracy.

**Table 5:** Comparative analysis of results obtained for Alzheimer's MRI dataset.

Model	Precision	Recall	F1-score	Accuracy	IoU	Dice	SSIM	MCC
GAN (base model)	0.701	0.529	0.603	0.715	0.462	0.632	0.584	0.561
GAN+L2	0.759	0.594	0.666	0.774	0.523	0.687	0.639	0.612
GAN+SSIM	0.793	0.653	0.716	0.809	0.567	0.724	0.672	0.648
Base Model+L2+SSIM	0.831	0.701	0.760	0.844	0.612	0.759	0.703	0.714
*f*_*g*_ +*f*_*d*_ + without skip connections	0.849	0.758	0.801	0.861	0.632	0.775	0.721	0.745
*f*_*g*_ +*f*_*d*_ + with skip connections	0.873	0.821	0.846	0.886	0.675	0.806	0.762	0.787
*f*_*g*_ +*f*_*d*_ + with skip connections + MLP	0.874	0.833	0.853	0.889	0.691	0.815	0.773	0.794
Proposed *S*^3^ Net	0.881	0.846	0.863	0.898	0.714	0.833	0.787	0.802

The addition of skip connections further boosts model performance. The *f*_*g*_ + *f*_*d*_ + U-Net with skip connections attains an F1-score of 0.846 and accuracy of 0.886. Replacing the Spiking Neural Network with an MLP classifier in the *f*_*g*_ + *f*_*d*_ + U-Net with skip connections framework achieves an F1-score of 0.853 and accuracy of 0.889, demonstrating that the proposed Spiking Network provides improved feature discrimination and classification performance compared to MLP based learning. Skip connections facilitate gradient flow and preserve fine spatial details. The proposed S^3^ Net achieves the highest performance. It records an F1-score of 0.863 and an accuracy of 0.898. Compared to the base GAN, this corresponds to approximately 26% improvement in F1-score and 18% in accuracy. Higher Dice (0.833) and SSIM (0.787) values confirm effective anatomical detail preservation and segmentation reliability.

### 5.3 Comparative analysis of results for MRI dataset

Table 6 summarizes the performance of the proposed *S*^3^ Net and its intermediate variants on the MRI dataset. The GAN base model serves as the baseline and achieves a precision of 0.707, a recall of 0.523, an F1-score of 0.601, and an accuracy of 0.719. These metrics indicate limited segmentation capability and weak feature representation. Combining the L2 loss (GAN+L2) model improves pixel-level accuracy and stability. This variant achieves a precision of 0.764, a recall of 0.597, the F1-score of 0.670, and an accuracy of 0.779. Adding SSIM loss further enhances structural preservation and segmentation quality. The GAN+SSIM model attains a precision of 0.798, a recall of 0.659, the F1-score of 0.722, and an accuracy of 0.814.

**Table 6:** Comparative analysis of results obtained for MRI dataset.

Model	Precision	Recall	F1-score	Accuracy	IoU	Dice	SSIM	MCC
GAN (base model)	0.707	0.523	0.601	0.719	0.458	0.628	0.581	0.557
GAN + L2	0.764	0.597	0.670	0.779	0.519	0.683	0.641	0.616
GAN + SSIM	0.798	0.659	0.722	0.814	0.564	0.721	0.675	0.652
Base model + L2 + SSIM	0.839	0.705	0.767	0.848	0.611	0.758	0.709	0.721
*f*_*g*_ + *f*_*d*_ + without skip connections	0.856	0.768	0.810	0.867	0.645	0.785	0.736	0.758
*f*_*g*_ + *f*_*d*_ + with skip connections	0.884	0.842	0.862	0.894	0.691	0.818	0.774	0.798
*f*_*g*_ + *f*_*d*_ + with skip connections + MLP	0.872	0.836	0.854	0.889	0.703	0.826	0.781	0.806
Proposed *S*^3^ Net	0.865	0.881	0.873	0.902	0.774	0.873	0.823	0.839

The combination of L2 and SSIM losses in the Base Model+L2+SSIM yields additional gains, reaching a precision of 0.839, recall of 0.705, F1-score of 0.767, and accuracy of 0.848. Feature fusion via *f*_*g*_ + *f*_*d*_ + U-Net without skip connections improves contextual understanding, achieving an F1-score of 0.810 and accuracy of 0.867. Incorporating skip connections enhances the model's ability to retain spatial details, with the *f*_*g*_ + *f*_*d*_ + U-Net with skip connections variant achieving an F1-score of 0.862 and accuracy of 0.894. Replacing the Spiking Neural Network with an MLP classifier in the *f*_*g*_ + *f*_*d*_ + U-Net with skip connections framework achieves a precision of 0.872, recall of 0.836, F1-score of 0.854, and accuracy of 0.889. The proposed *S*^3^ Net achieves the highest performance among all models. It records a precision of 0.865, a recall of 0.881, a F1-score of 0.873, and an accuracy of 0.902. Additionally, it attains the best scores for IoU (0.774), Dice coefficient (0.873), SSIM (0.823), and MCC (0.839), demonstrating that the proposed Spiking Network improves classification robustness and segmentation-aware feature learning over conventional MLP-based classification. The obtained results prove that the *S*^3^ Net model accurately preserves structural details and provides reliable segmentation for the MRI dataset.

**Figure 13:**
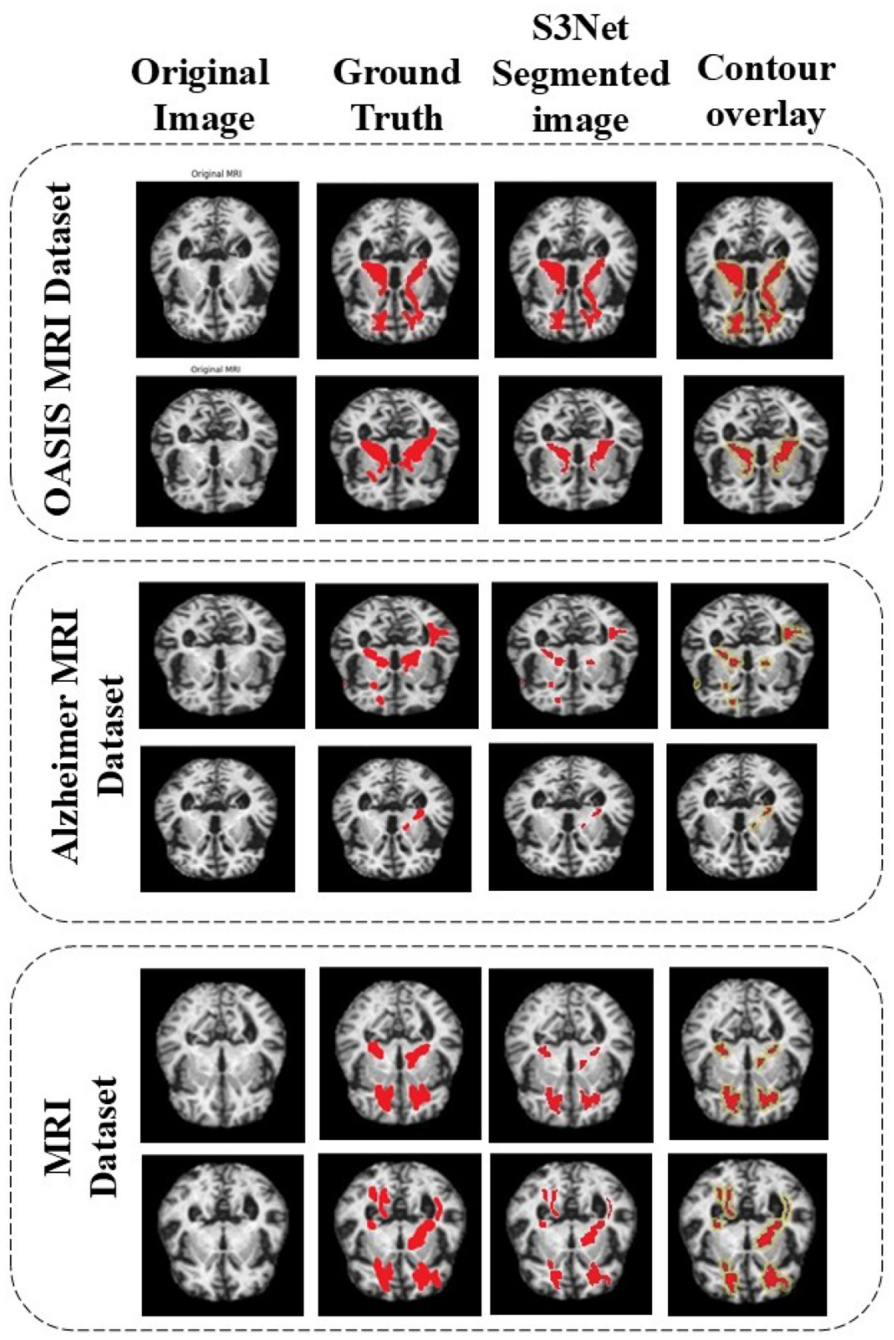
Illustrates the semantic segmentation and classification results for Alzheimer's disease across three datasets. The first, second, and third dataset results are shown in successive row pairs, followed by a contour overlay comparing ground-truth (yellow) and predicted (green) boundaries.

The Figure 13 represents a sample of semantic segmentation and image classification results for AD across three datasets. The first two rows display segmented regions alongside their corresponding classified images from the OASIS dataset. The next two rows present the segmented regions and classified images from the Alzheimer's MRI dataset, followed by two rows showing the same results for the general MRI dataset. Finally, a contour overlay is provided to compare the ground-truth boundaries (yellow) with the model's predicted boundaries (green), demonstrating the segmentation accuracy.

### 5.4 Statistical comparative analysis across datasets

Tables 7–9 present the statistical evaluation of the proposed *S*^3^ Net and its intermediate variants on the OASIS, Alzheimer's, and general MRI datasets, respectively. The metrics reported include mean accuracy, F1-score, MCC, 95% confidence intervals, and associated *p*-values. The reported standard deviations and 95% confidence intervals are computed using a test set bootstrapping strategy. After the final model training phase, the test set predictions is resampled with replacement for 1,000 bootstrap iterations. For each bootstrap iteration, evaluation metrics including accuracy, F1-score and MCC were recomputed. The mean and standard deviation values were subsequently calculated across all bootstrap samples. Furthermore, the 95% confidence intervals were estimated using the percentile based bootstrap method, thereby improving the statistical reliability and reproducibility of the reported results. For all datasets, the GAN base model exhibits the lowest performance. On the OASIS dataset (Table 7), it achieves an accuracy of 0.741 ± 0.028 and F1-score of 0.627 ± 0.025. The Alzheimer's dataset (Table 8) shows slightly lower accuracy (0.715 ± 0.030) and F1-score (0.603 ± 0.026). Similarly, the general MRI dataset (Table 9) achieves an accuracy of 0.719 ± 0.028 and F1-score of 0.601 ± 0.026. These results indicate that the base GAN provides limited feature representation and unstable segmentation across datasets.

**Table 7:** Statistical comparison of model performance on OASIS MRI dataset.

Model	Accuracy (mean ±SD)	95% Confidence interval (accuracy ±SD)	F1-score (mean ±SD)	MCC (mean ±SD)	*P*-value
GAN (base model)	0.741 ± 0.028	(0.713 ± 0.028–0.769 ± 0.028)	0.627 ± 0.025	0.579 ± 0.027	0.042
GAN + L2	0.792 ± 0.024	(0.768 ± 0.024–0.816 ± 0.024)	0.686 ± 0.022	0.632 ± 0.023	0.033
GAN + SSIM	0.832 ± 0.021	(0.811 ± 0.021–0.853 ± 0.021)	0.741 ± 0.020	0.675 ± 0.021	0.028
Base model + L2 + SSIM	0.861 ± 0.019	(0.842 ± 0.019–0.880 ± 0.019)	0.777 ± 0.018	0.731 ± 0.019	0.021
*f*_*g*_ +*f*_*d*_ + without skip connections	0.883 ± 0.017	(0.866 ± 0.017–0.900 ± 0.017)	0.825 ± 0.015	0.775 ± 0.017	0.015
*f*_*g*_ +*f*_*d*_ + with skip connections	0.923 ± 0.012	(0.911 ± 0.012–0.935 ± 0.012)	0.899 ± 0.010	0.841 ± 0.012	0.004
*f*_*g*_ +*f*_*d*_ + with skip connections + MLP	0.931 ± 0.011	(0.920 ± 0.011–0.942 ± 0.011)	0.910 ± 0.009	0.854 ± 0.011	0.003
Proposed *S*^3^ Net	**0.951 ± 0.010**	**(0.941 ± 0.010–0.961 ± 0.010)**	**0.930 ± 0.008**	**0.893 ± 0.010**	< 0.001

**Table 8:** Statistical comparison of model performance on Alzheimer's MRI dataset.

Model	Accuracy (mean ±SD)	95% Confidence interval (accuracy ±SD)	F1-score (mean ±SD)	MCC (mean ±SD)	*P*-value
GAN (Base model)	0.715 ± 0.030	(0.685 ± 0.030–0.745 ± 0.030)	0.603 ± 0.026	0.561 ± 0.029	0.044
GAN + L2	0.774 ± 0.027	(0.747 ± 0.027–0.801 ± 0.027)	0.666 ± 0.023	0.612 ± 0.025	0.037
GAN + SSIM	0.809 ± 0.023	(0.786 ± 0.023–0.832 ± 0.023)	0.716 ± 0.021	0.648 ± 0.023	0.029
Base Model + L2 + SSIM	0.844 ± 0.020	(0.824 ± 0.020–0.864 ± 0.020)	0.760 ± 0.018	0.714 ± 0.020	0.022
*f*_*g*_ + *f*_*d*_ + without skip connections	0.861 ± 0.018	(0.843 ± 0.018–0.879 ± 0.018)	0.801 ± 0.016	0.745 ± 0.018	0.015
*f*_*g*_ + *f*_*d*_ + with skip connections	0.886 ± 0.016	(0.870 ± 0.016–0.902 ± 0.016)	0.846 ± 0.013	0.787 ± 0.015	0.009
*f*_*g*_ + *f*_*d*_ + with skip connections + MLP	0.889 ± 0.014	(0.875 ± 0.014–0.903 ± 0.014)	0.853 ± 0.012	0.794 ± 0.013	0.005
Proposed *S*^3^ Net	0.898 ± 0.013	(0.885 ± 0.013–0.911 ± 0.013)	0.863 ± 0.011	0.802 ± 0.013	< 0.001

**Table 9:** Statistical comparison of model performance on MRI dataset.

Model	Accuracy (mean ±SD)	95% Confidence interval (accuracy ±SD)	F1-score (mean ±SD)	MCC (mean ±SD)	*P*-value
GAN (base model)	0.719 ± 0.028	(0.691 ± 0.028–0.747 ± 0.028)	0.601 ± 0.026	0.557 ± 0.027	0.042
GAN + L2	0.779 ± 0.025	(0.754 ± 0.025–0.804 ± 0.025)	0.670 ± 0.023	0.616 ± 0.024	0.036
GAN + SSIM	0.814 ± 0.021	(0.793 ± 0.021–0.835 ± 0.021)	0.722 ± 0.020	0.652 ± 0.022	0.027
Base model + L2 + SSIM	0.848 ± 0.019	(0.829 ± 0.019–0.867 ± 0.019)	0.767 ± 0.018	0.721 ± 0.019	0.020
*f*_*g*_ + *f*_*d*_ + without skip connections	0.867 ± 0.017	(0.850 ± 0.017–0.884 ± 0.017)	0.810 ± 0.016	0.758 ± 0.017	0.015
*f*_*g*_ + *f*_*d*_ + with skip connections	0.894 ± 0.014	(0.880 ± 0.014–0.908 ± 0.014)	0.862 ± 0.013	0.798 ± 0.014	0.008
*f*_*g*_ + *f*_*d*_ + with skip connections + MLP	0.889 ± 0.015	(0.874 ± 0.015–0.904 ± 0.015)	0.854 ± 0.012	0.806 ± 0.013	0.005
Proposed *S*^3^ Net	0.902 ± 0.012	(0.890 ± 0.012–0.914 ± 0.012)	0.873 ± 0.011	0.839 ± 0.012	< 0.001

Incorporating the L2 loss consistently improves performance. The GAN+L2 model increases accuracy by approximately 5–6% over the base GAN on all datasets. F1-scores also improve by roughly 6—7%, indicating enhanced pixel-level reconstruction. Incorporating SSIM loss further strengthens structural consistency. The GAN+SSIM variants reach accuracies of 0.832, 0.809, and 0.814 for OASIS, Alzheimer's, and general MRI datasets, respectively. F1-scores improve proportionally, reflecting better preservation of anatomical structures. Combining L2 and SSIM losses yields additional gains. The Base Model + L2 + SSIM achieves accuracies of 0.861, 0.844, and 0.848, and F1-scores of 0.777, 0.760, and 0.767, respectively. Feature fusion using *f*_*g*_ + *f*_*d*_ + U-Net without skip connections improves contextual understanding, increasing F1-scores to 0.825, 0.801, and 0.810. Including skip connections further enhances spatial feature retention. The *f*_*g*_ + *f*_*d*_ + U-Net with skip connections reaches accuracies of 0.923, 0.886, and 0.894, with F1-scores of 0.899, 0.846, and 0.862, respectively.

Replacing the Spiking Neural Network with an MLP classifier in the *f*_*g*_ + *f*_*d*_ + U-Net with skip connections framework achieves accuracies of 0.931, 0.889 and 0.889, with corresponding F1-scores of 0.910, 0.853 and 0.854 for the OASIS, Alzheimer's MRI and MRI dataset, respectively. Although the MLP based framework improves classification performance over the conventional fusion models, the proposed Spiking Neural Network consistently achieves superior accuracy, F1-score, and MCC across all datasets.

The proposed *S*^3^ Net achieves the highest and most consistent performance across all datasets. On OASIS, it achieves 0.951 ± 0.010 accuracy and 0.930 F1-score. On Alzheimer's MRI, it reaches 0.898 ± 0.013 accuracy and 0.863 F1-score. On the general MRI dataset, accuracy is 0.902 ± 0.012 and F1-score 0.873. MCC values also increase consistently, confirming reliable prediction quality. *P*-values are all below 0.001, indicating statistically significant improvements over baseline and intermediate variants. These results confirm that *S*^3^ Net robustly preserves anatomical details and consistently delivers high-quality segmentation across multiple MRI datasets.

### 5.5 Comparative analysis of other state-of-the-art models

The proposed *S*^3^ Net model shows strong and consistent performance when compared with other state-of-the-art methods as indicated in Table 10. It achieves a precision of 0.942, recall of 0.918, and an F1-score of 0.930, showing that it can correctly detect important features while keeping errors low. Its accuracy of 0.951 is also higher than models such as DenseNet121 (Kubi and Nazir, 2025) and the Genetic Algorithm approach (Al-Najdawi et al., 2025). The model's IoU (0.826) and Dice score (0.905) indicate very reliable segmentation results, performing better than the FED-UNet++ model (Yang et al., 2025). In addition, the strong SSIM (0.879) and MCC (0.893) values show that *S*^3^ Net preserves structural details well and makes stable predictions. When compared with other studies, many existing models perform well only in certain areas but not across all metrics. For instance, Automatic MRI Segmentation Techniques (Bonarota et al., 2025) and HybridCA-Net (Dakshinamoorthy et al., 2025) achieve very high recall scores but do not report precision or F1-scores, making it difficult to judge their overall performance. GLA-GAN (Sikka et al., 2025) shows excellent SSIM but has a lower recall, meaning it may miss some important regions. In contrast, the proposed *S*^3^ Net delivers strong results across every major metric, making it more reliable and effective overall compared to the models listed in Bonarota et al. (2025), Kadri et al. (2025), Kubi and Nazir (2025), and Yang et al. (2025).

**Table 10:** Comparative analysis of results obtained for other state-of-the-art models.

Model (dataset)	Precision	Recall	F1-score	Accuracy	IoU	Dice	SSIM	MCC
Automatic MRI segmentation techniques (Bonarota et al., 2025) (ADNI, memory clinics)	–	0.970	–	0.940	–	–	–	–
HybridCA-Net (Dakshinamoorthy et al., 2025) (ADNI sMRI + fMRI)	–	0.9734	–	0.9419	–	–	–	-
Genetic algorithm (Al-Najdawi et al., 2025) (TCIA)	0.86	0.88	0.87	–	–	–	–	–
DenseNet121 (Kubi and Nazir, 2025) (Dementia clinical dataset)	0.85	0.60	0.87	0.90	–	–	–	–
Multi-order 3D Unet (Sun et al., 2024) (179 Alzheimer's MRI)	0.843	0.870	–	–	0.759	–	–	–
GLA-GAN (Sikka et al., 2025) (ADNI paired MRI + FDG-PET)	–	0.825	0.825	0.829	–	–	0.969	–
FED-UNet++ (Yang et al., 2025) (Hippocampus benchmark)	0.969	0.898	–	0.986	0.825	0.903	–	–
Proposed *S*^3^ Net (OASIS)	0.942	0.918	0.930	0.951	0.826	0.905	0.879	0.893

## 6 Conclusion

In this study, a novel Synthesis–Segmentation–Spiking Network (*S*^3^-Net) is proposed for automated AD detection and lesion segmentation from MRI scans. The framework comprises three sub-networks: (1) a Synthesis Network that generates pathology-aware synthetic MRI images using GANs to enhance structural consistency, (2) a Segmentation Network that fuses generator and discriminator feature maps within an encoder–decoder architecture for precise lesion delineation, and (3) a Spiking Network that performs energy-efficient classification using temporal spike dynamics. By fusing latent representations from the synthesis and segmentation modules, *S*^3^-Net effectively captures both global anatomical and local lesion-specific features, improving segmentation accuracy and disease classification. Comprehensive evaluation on three benchmark datasets—OASIS, Alzheimer's MRI, and a General MRI dataset—demonstrates the robustness and generalization capability of the proposed model. *S*^3^-Net achieves an Accuracy of 95.1%, F1-score of 93.0%, and IoU of 89.3% on the OASIS dataset; an Accuracy of 89.8%, F1-score of 86.3%, and Dice coefficient of 83.3% on the Alzheimer's MRI dataset; and an Accuracy of 90.2%, F1-score of 87.3%, and IoU of 77.4% on the General MRI dataset. These results confirm that *S*^3^-Net significantly outperforms existing GAN-based and hybrid approaches in terms of segmentation precision, structural preservation, and classification reliability. Although the proposed *S*^3^Net achieved promising performance for MRI-based AD analysis, clinical validation using diagnostic indicators such as CDR scores, MMSE assessments, and CSF biomarkers was not performed in this study. This will be addressed in future work, along with extending *S*^3^Net to multi-modal MRI fusion to capture complementary anatomical and pathological features, incorporating cost-sensitive learning to address class imbalance, and exploring transformer-based architectures to improve contextual reasoning and scalability for clinical deployment.

## Data Availability

The raw data supporting the conclusions of this article will be made available by the authors, without undue reservation.
